# Investigating extreme hydrological risk impact on water quality; evidence from Buffalo catchment headwater, Eastern Cape, South Africa

**DOI:** 10.1007/s11356-023-27048-4

**Published:** 2023-05-16

**Authors:** Solomon Temidayo Owolabi, Johanes A. Belle

**Affiliations:** Disaster Management Training and Education Centre for Africa, Faculty of Natural and Agricultural Sciences, University of the Free State, P. O. Box 339, Bloemfontein, 9300 Free State South Africa

**Keywords:** Climate change, Hydrological drought, Hydrochemistry, Confusion matrices, Spatio-temporal assessment, Wavelet coherence

## Abstract

Evidence from increasing mineralization, micropollutant concentrations, waterborne epidemics, an algal boom, and dissolved organic matter has provided substantial evidence that climate change impacts water quality. While the impact of the extreme hydrological event (EHE) on water quality (WQ) has aroused considerable research interest, research uncertainty has been premised on WQ data scarcity, a short time frame, data non-linearity, data structure, and environmental biases on WQ. This study conceptualized a categorical and periodic correlation using confusion matrices and wavelet coherence for varying standard hydrological drought index (SHDI; 1971–2010) and daily WQ series (1977–2011) of four spatially distinct basins. By condensing the WQ variables using chemometric analyses, confusion matrices were assessed by cascading the SHDI series into 2-, 3-, and 5-phase scenarios. 2-phase revealed an overall accuracy (0.43–0.73), sensitivity analysis (0.52–1.00), and Kappa coefficient (− 0.13 to 0.14), which declines substantially with the phase increase, suggesting the disruptive impact of EHE on WQ. Wavelet coherence depicted the substantial (${R}_{n}^{2}\left(u,s\right)\ge 0.5$) mid- and long-term (8–32 days; 6–128 days) co-movement of streamflow over WQ, confirming the varying sensitivity of WQ variables. Land use/land cover mapping and the Gibbs diagram corroborate the eventful WQ evolution by EHE and their spatial variability concerning landscape transformation. Overall, the study deduced that hydrologic extreme triggers substantial WQ disruption with dissimilar WQ sensitivity. Consequently, suitable chemometric indicators of EHE impacts such as WQ index, nitrate-nitrogen, and Larson index at designated landscapes were identified for extreme chemodynamics impact assessment. This study proffers a recommendation for monitoring and managing the impact of climate change, floods, and drought on water quality.

## Introduction

The recent increase in the periodicity of extreme hydrological events (EHE) shows a profound impact on nutrients loads of water quality (WQ), thus compromising river health and altering ecosystem services (Keener et al. 2010; Afuye et al. 2022). The flood (drought) event triggered by the La Niña (El Niño) phase of the El Niño Southern Oscillation is a period of prolonged wetness (dryness), and it results in heavy overbank stormflow (diminution) (Blamey et al. 2018; Pomposi et al. 2018). Consequently, the excesses (deficit) in water flow accelerates (truncates) the dilution rates of point source emissions and lowers (increases) the residence time of concentration loads, though inefficiently for a flash flood (Peña-Guerrero et al. 2020). The impact of the two phases of EHE has been linked with a sudden rise in water reservoir contamination and waterborne diseases (Van Vliet and Zwolsman 2008; Cann et al. 2013; Logan et al. 2022; Chow et al. 2023). This further compounds the projected influence of increasing anthropogenic activities on the sustainable development of the environment (Change 2014; Barnett et al. 2015). The extent of water quality alteration depends solely on geology, land use, and land cover system as the source of the agent causation (Van Loon 2015; Namugize et al. 2018; Mararakanye et al. 2022). Population increase, urbanization rate, and socioeconomic activities are the land use/land cover system that stresses WQ on the short- and long-term evolution of water quality (Vogt et al. 2016; Mararakanye et al. 2022). Also, environmental geology is a natural long-term point source of water contamination. However, the significant impact of EHE as event causation has been noted by the Intergovernmental Panel on Climate Change, as marked by its negative consequence on the global economic system (Change 2014; Borrelli et al. 2020; Masson-Delmotte et al. 2021). Studies on EHE-concentrated loading interplay are vital for river and environmental health management.

Several pieces of research have contributed to understanding water quality evolution in response to extreme hydro-climatic events. To this end, applying a probabilistic vulnerability assessment approach substantiates the impact of drought on specific WQ variables (Kim et al. 2019). As a result of the increased residence time, a drought event induces biogeochemical shifts, anaerobic metabolism, and hypoxic conditions critical to the development of eutrophication and waterbody stratification (Wright et al. 2014; Eslamian and Eslamian 2017; Gómez-Gener et al. 2020). Mishra et al. (2021) reported nutrient loading (total phosphorus and nitrogen), inverse synchrony, and mutual relativity of point source facilities with drought events. Contrarily, an empirical assessment of unbalanced panel data from 62 countries showed that floods have a more adverse impact on water quality (Zou et al. 2022). While both the floods and drought events produce an evolutionary change in WQ identity (Change 2014; Konapala et al. 2020), it is still difficult to substantiate the sensitivity of water chemical species and water facie to different phases of hydrologic extremes.

The geographical location of Buffalo headwater catchment (BHC) at the transitional zone of the subtropical and monsoon climates of South Africa makes it a worthy reference point for this study due to its cataclysmic flood and drought events (Jury 2015; Mahlalela et al. 2020; Owolabi et al. 2022). Three distinct landscape settings delineate BHC: natural, semi-natural, and artificial (Owolabi et al. 2020b). Due to prolonged dryness, the river recurses to dependency on baseflow (Owolabi et al. 2020a), and this further aggravates the impact of the effluent discharge from a wastewater treatment plant proximal to the mouth of the catchment (Chigor et al. 2013; Edokpayi et al. 2017). Meanwhile, South Africa’s municipal waste management authority and water resource management have been faced with huge setbacks regarding the provision of safe and clean water in 2010. This is due to the collapse of about 328 of the 824 wastewater treatment plants in South Africa and the flow of raw and partially treated sewage into the river system (Odendaal 2017; Herbig 2019). The protection of South Africa’s rivers, pollution control, and waste water management are backed by two legal frameworks: the National Water Acts (NWA – Act 36 of 1998) and the National Environmental Management Act, 1998 (Herbig 2019). The water services authorities are responsible for the provision of water and sanitation services according to the national legislation: the South African Constitution, 1996 (Act 108 of 1996), the Municipal Structures Act, 1998 (Act 117 of 1998), the Municipal Systems Act, 2000 (Act 32 of 2000), and the Water Services Act of 1997 (Act 108 of 1997).

To this end, several calls have been made for research on water quality trends. Previous studies within the study region were generally focused on the short-term dispersivity of WQ. To the author’s best knowledge, no study has considered the probable long-term effects of EHE on the suitability of surface water due to its substantial water provision to more than 200,000 dwellers within the area (Owolabi et al. 2020b; Nolte et al. 2021). Comparative regional studies were focused on drought risk of pansteatitis spread (Dabrowski et al. 2014), calibration of WQ deterioration rate due to drought (Mhlongo et al. 2018), land use/land cover change impact on river health (Mararakanye et al. 2022), and drought impact on WQ dynamics using 166 diatoms (Holmes et al. 2022). So far, most of the investigations have been limited to the holistic extreme hydrological event and its consequences. Moreover, the selection of appropriate comparative methods was not thoroughly ratified, considering EHE’s periodic and categorical properties.

This study’s novelty lies in assessing the impact of varying EHE phases and scenarios on water suitability indices using categorical and periodic correlation, as well as tailoring the assessment to specific environmentally cogent questions. The categorical evaluation of the water quality impact of EHE is achieved using confusion matrices, while the periodic correlation was undertaken within the time–frequency domain of wavelet coherence analysis. The assessment takes into account the dual geology terrain's spatial heterogeneity in landscape integrity. Confusion matrices (CM) have provided valuable insights into the drought scenerio for onion species productivity (Ropelewska et al. 2022), agricultural drought index evaluation (Hazaymeh and Hassan 2017), fluoride-contaminated water suitability analysis (Barad et al. 2021), and drought model validation (Kolachian and Saghafian 2021). Moreover, the ramifications of wavelet coherence (WC) as a magnitude quantifier of nonparametric correlation of environmental variables and hydrological studies have been proven enormously in the literature (Torrence and Compo 1998; Coulibaly and Burn 2004; Tamaddun et al. 2017; Owolabi et al. 2022). The hybrid approach of confusion matrices and wavelet coherence analysis as a periodic and categorical regressor of water quality and the EHE relationship has not been employed anywhere else. In this study, the transmutation of CM and WC is intended to address the following questions:

i. To what extent do extreme hydrological events impact surface water quality and its suitability for drinking and agricultural activities?
ii. How sensitive are water integrity and its suitability to varying levels of extreme hydrological events?
iii. How does the risk impact of drought intensity on surface water suitability vary under different landscape categories?

The current study intends to explore the impact of extreme hydrological events on surface water quality and its suitability. To this end, the study projects the drought variability across some selected basins of BCH, representing three distinct landscapes. It cascades the EHE into levels to obtain three scenarios of climatic events for scalar assessment with WQ indices. Chemometric analyses based on water suitability were employed to reduce the volume of WQ components and the computation ambiguity (Huang and Liu 2008; Sun et al. 2012). These include the water quality index, Larson and Skold index, Ryznar stability index, sodium adsorption ratio, and exchangeable sodium. These were investigated for their trends alongside physicochemical parameters, total dissolved solids, electrical conductivity, and total nitrates. The study is further supported by the inspection streamflow co-movement on the selected WQ indices and corroborated by land use/land cover mapping and Gibbs plot.

## Study site

**Fig. 1:**
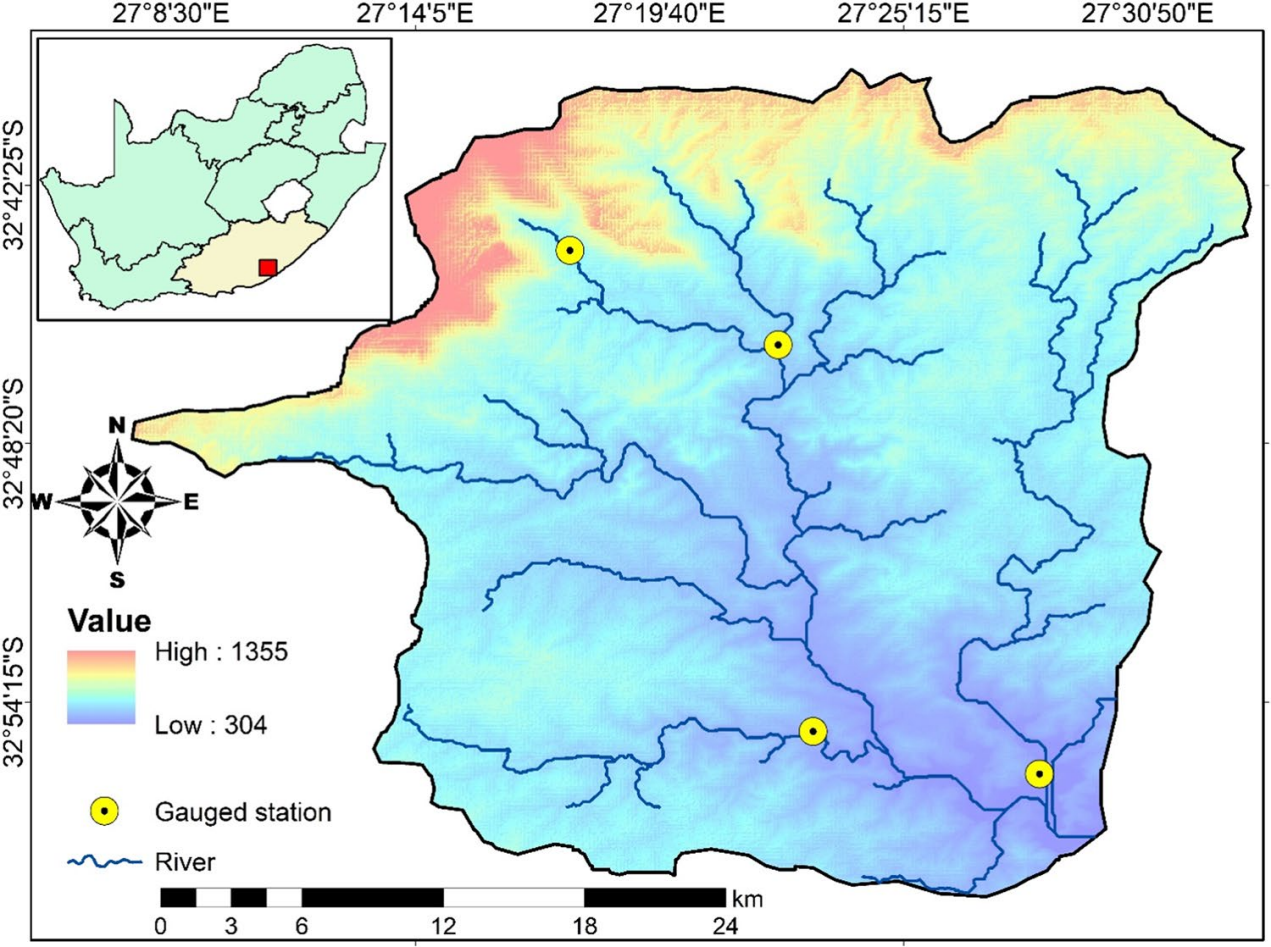
The location of Buffalo catchment headwater and the hydrometric stations

Buffalo catchment headwater (BCH) is located on the southern coast of Eastern Cape in South Africa, within latitude 32°39′ to 33°05′ S and longitude 27°05′ to 27°33′ E, covering approximately 1237 km^2^ (Fig. 1). BCH has a complex terrain with an altitudinal range of 250–1370 m.a.s.l. and three distinct landforms, the medium gradient mountain (30%), the dissected plain (42%), and the plain (28%) (Owolabi et al. 2020a). BCH is a dendritic drainage, with the central river valley, Buffalo River, running southeasterly into the Indian Ocean, about 126 km long, from Isidenge nature forest. The headwater divides across six major sub-catchments; Quencwe, Zelitsha North, Mgqakwebe, Tshoxa, Ngqokweni, and Yellowwoods watersheds. The hydro-climatic attribute of BCH is bimodal, exhibiting the subtropical (easterly) and the monsoon (westerly) climate (Owolabi et al. 2022). Thus, it varies temporally and spatially across its terrain, with a mean annual rainfall of 590 mm and a range of 332 mm and 844 mm in the driest and wettest years between 1987 and 2017, while its yearly mean temperature is 13.5 °C and 22.3 °C in winter and summer respectively (Owolabi et al. 2021a).

The hydro-climatic events vary in intensity across the landform with occasional extremity at the hilly terrain (Owolabi et al. 2020a). Hence, the basin is characterized by three main eco-topographic biomes dominated by the dense forest at the hilly terrain, the thicket at the dissected plain, and the grassland at the plain (Owolabi et al. 2020b). The hilly landform is a part of the hinterland mountain range resulting from rift and subsidence induced by dolerite emplacement in the Karoo Supergroup in the Jurassic (Baiyegunhi et al. 2017). The geological components of the terrain include the Tatarian arenaceous mudstone of the Balfour Formation, the Kazarian argillaceous shaly-silty sandstone of the Middleton Formation, and Quaternary sediments, and the dolerite sills and dykes (Johnson et al. 2006). BCH groundwater is hosted by a fissured and intergranular aquifer (Owolabi et al. 2021b). The study is designed around the Upstream, Quencwe, Ngokweni, and Yellowwoods basins, which provide the three varying landscape attributes: the natural, semi-natural, and artificial landscapes (Table 1) and hydrometric stations for daily streamflow (1947–2021) and water quality (1977–2011) data.

**Table 1:** Description of Buffalo sub-catchment location, size, and landscape extent

Catchment	Station ID		Station (^○^)		Landscape type (%)			Area
Class	Flow	Sampling	Latitude	Longitude	Natural	Semi-natural	Artificial	(km^2^)
Upstream	R2H001	R20 102,507	− 32.73	27.29	96.1	0.2	3.7	26
Quencwe	R2H008	R20 102,512	− 32.77	27.37	78.4	15.8	5.8	142
Ngqokweni	R2H009	R20 102,513	− 32.92	27.39	4.5	57.0	38.6	105
Yellowwoods	R2H011	R20 102,515	− 32.93	27.47	35.7	33.9	30.4	187

The Department of Water Affairs (DWA) of South Africa provided the daily streamflow and water quality data. The water quality data collated explicitly included for this assessment are total dissolved solids (TDS), electrical conductivity (EC), hydrogen ion concentration (pH), chloride (CL), nitrate (NO_3_-NO_2_), sulfate (SO_4_), carbonate (HCO_3_), bicarbonate (CO_3_), magnesium (Mg), sodium (Na), potassium (K), total alkalinity, and calcium (Ca^2+^). The water quality monitoring and data collection were carried out and stored at random frequencies, varying in weekly, fortnightly, monthly, bimonthly, and biannually from 1977 in the DWA website (https://www.dws.gov.za/iwqs/wms/data/WMA12_reg_WMS_nobor.htm) (DWS 2019). Due to the little consideration for industrial activities, heavy metals were not factored into data collection and were not the focus of this study (DWAF 1996). Also, the DWA website (https://www.dws.gov.za/Hydrology/Verified/HyStations.aspx?Region=R&StationType=rbRiver) provided the streamflow data.

The standard hydrological drought index (SHDI) time series at 1-month (SHDI-1), 3-month (SHDI-3), and 12-month (SHDI-12) timescale were calculated using the maximum monthly discharge from the hydrometric station at the sub-catchment outlets. The water quality data volume is reduced into five chemometric analyses, which include Larson and Skold index (LI), Ryznar stability index (RSI), sodium adsorption ratio (SAR), exchangeable sodium percentage (ESP), and water quality index (WQI). Although the streamflow data of the four stations are uniform and consistent for the computation of the standard hydrological drought index, the physicochemical parameters of the four hydrometric stations were not sampled uniformly.

## Materials and methods

### Streamflow data

The study was achieved using daily streamflow data for four gauging stations in Buffalo. The decision on the selected length of the streamflow data set, 1971 to 2011, was due to the size of the water quality serving as the reference data in this study. Importantly, the study ensured the exactness of streamflow data with the water quality series irrespective of the weekly, biweekly, and discontinuity in the water quality time series to ensure the uniformity of the data set before the individual correlation assessment. The water quality time series runs from 10^th^ November 1973 to 06^th^ April 2011, 06^th^ September 1977 to 07^th^ February 2011, 15^th^ Jun 1974 to 11^th^ January 2011, and 15^th^ June 1974 to 14^th^ October 2003 with 597, 488, 429, and 611 degrees of freedom (dF) for Buffalo Upstream, Quencwe, Ngqokweni, and Yellowwood catchments respectively. Since the study objectives are not structured on a specific timeframe, the inconsistency in catchment scenarios cannot affect the study outcome. Moreover, the correlation assessment is satisfied with sufficient data length and adequate time confidence interval to address the correlation process variation and concerns associated with static timing (Agarwal et al. 2002; Yang et al. 2016). The streamflow data was thoroughly inspected for missing records, which were fewer than 5% of the entire series, and corrected using sequential indicator simulation (Juang et al. 2004; Lepot et al. 2017).

### Standard hydrological drought index

The standard drought estimation approach recommended by the world Meteorological Organization was proposed by McKee et al. (1993) and is known as the standard precipitation index (SPI). As SPI applies to meteorological drought estimation, the standard hydrological drought index (SHDI) is deployed for hydrological drought investigation. Importantly, SHDI is computed similarly to SPI, while steps such as the computation and fitting of the presumed probability density function and the cumulative distribution function were skipped (Dehghani et al. 2014; Kolachian and Saghafian 2021). Hence, its computation is described in Eq. 1:

1 $$SHDI=\frac{{q}_{ij}-{q}_{im}}{\sigma }\times 100$$

where *q*_*ij*_ is the seasonal streamflow at the *i*^th^ gauging station and *j*^th^ observation, *q*_*im*_ is the long-term seasonal mean flow, and σ is its standard deviation. SHDI is presumed to be equivalent to the *z*-value of the normal distribution as it applies to SPI. Hence, McKee et al. (1993) proposed the following classification: extreme wetness (*z* > 2.0), high wetness (2.0 ≤ *z* < 1.5), moderate wetness (1.5 ≤ *z* < 1.0), near normal (1.0 ≤ *z* <  − 1.0), moderate drought (− 1.0 ≤ *z* <  − 1.5), severe drought (− 1.5 ≤ *z* <  − 2.0), and extreme drought (*z* ≤  − 2.0).

The SHDI computation was performed in RStudio using the standardized precipitation-evaporation index (SPIE) package, v.1.7, designed by Vicente-Serrano et al. (2010). The daily series were summed into monthly streamflow data, and the SPI-1 was computed to provide the effective monthly drought intensity required for the drought-concentrated loading comparative analysis. Also, SPI-3 and SPI-12 were obtained to project the temporal variation of hydrological drought across the investigated time frame (October 1971 to March 2011).

### Water quality data

In situ water quality sampling for physicochemical parameters such as pH, TDS (mg/L), and EC (μS/cm) was analyzed in the field with a two-point calibration radiometer (TTT85), turbidity meter, and pH meter in the early years until 1995 when more advances probe/loggers were employed, while others, including the hydro-chemical analyses, were performed in the Talbot laboratories, Port Elizabeth, Eastern Cape, South Africa, and others recommended by the Department for Water and Sanitation (DWS 2020). The analytical procedures for assessing the inorganic determinants were detailed in Van Vliet et al. (1988), van Vuuren and Pieterse (2005) and varied across the timeline. However, they are documented in the analytical methods manuals TR136 and TR151 at http://www.dwa.gov.za/iwqs/reports/tr.aspx. The ionic balance for each periodic row of the selected time series is less than 5%, while the calculated total dissolved solids (TDS) was 99.8% identical to the in situ TDS for all six stations. The missing data was less than 0.5%. The calculated TDS filled in the missing TDS data, while the missing time row with significant inconsistency was not considered. Other notable uses of the water quality data for research purposes include Du Plessis et al. (2014), Griffin et al. (2014), Griffin (2017), Namugize et al. (2018), Banda and Kumarasamy (2020), and Mararakanye et al. (2022).

#### Chemometric analyses

Chemometric analyses based on LI and RSI quantify the environmental flow’s degree of degradation and corrosiveness (Larson and Skold 1958; Nabila 2014). To obtain this, the molarity ratio of sulfate and chloride to bicarbonate was estimated as presented in Eq. 2:

2 $$LI=\frac{\left[{\mathrm{SO}}_{4}^{2-}\right]+[{\mathrm{Cl}}^{-}]}{[{\mathrm{HCO}}_{3}^{-}-{\mathrm{CO}}_{3}^{2-}]}$$

where *LI* is the Larson index, [SO_4_^2−^], [Cl^−^], [HCO_3_^−^], and [CO_3_^2−^] are the concentration of sulfate, chloride, bicarbonate, and carbonate measured in mmol/L. The LI time series was also computed for further statistical analysis to indicate water quality evolution/freshness. The following categorization applies: LI < 0.8, scaling tendency; 0.8 ≤ LI < 1.2, corrosive; and LI > 1.2, strongly corrosive (Yousefi et al. 2016). However, RSI was factored into this study due to the predisposition for calcium ion enrichment and the tendency to form scale and otherwise as a predictive tool where corrosivity may arise. RSI computations are presented according to Eq. 3:

3 $$RSI={2\mathrm{pH}}_{s}-\mathrm{pH}$$

pH is the substantial degree of salinity and acidity measured in the field, while pH is calculated using Eq. 4–8.

4 $${\mathrm{pH}}_{s}=\left(9.3+A+B\right)-(C+D)$$

5 $$A=\frac{[{\mathrm{Log}}_{10}\left(TDS\right)-1]}{10}$$

6 $$B=-13.12 \times {\mathrm{Log}}_{10}\left(T^\circ C+273\right)+34.55$$

7 $$C={\mathrm{Log}}_{10}\left({\mathrm{Ca}}^{2+}\mathrm{as\;CaC}{\mathrm{O}}_{3}\mathrm{ mg}/\mathrm{L}\right)-0.4$$

8 $$D={\mathrm{Log}}_{10}\left(\mathrm{Alkalinity\;as\;CaC}{\mathrm{O}}_{3}\mathrm{\;mg}/\mathrm{L}\right)$$

where *TDS* is the total dissolved solids measured in the field, *T°C* is the in situ water temperature, and Ca^2+^ is the calcium ion. The RSI deductions categorized the nature of water into the following criteria: strongly encrusting (3 ≤ RSI < 5); supersaturated and moderately encrusting (5 ≤ RSI < 6); saturated and low encrusting (6 ≤ RSI < 7); under-saturated with mild corrosiveness (7 ≤ RSI < 7.5); strongly corrosive (7.5 ≤ RSI < 9); and highly corrosive (RSI ≥ 9) (Yousefi et al. 2016).

The assessment of SAR and ESP guides the surface water suitability for irrigation purposes. High SAR and ESP are detrimental to the soil structure as it reduces the hydraulic conductivity of the soil (El Bilali and Taleb 2020). SAR and ESQ are estimated using Eqs. 9 and 10:

9 $$ESP= \frac{\mathrm{Exchangeable\;}{\mathrm{Na}}^{+}}{{\mathrm{Ca}}^{2+}+{\mathrm{Mg}}^{2+}+{\mathrm{K}}^{+} + {\mathrm{Na}}^{+}+{\mathrm{Al}}^{+} +\mathrm{ H}}\times 100$$

10 $$SAR= \frac{{\mathrm{Na}}^{+} }{\sqrt{\frac{{\mathrm{Mg}}^{2+}+ {\mathrm{Ca}}^{2+}}{2}}}$$

Buffalo water’s suitability for drinking was premised on calculating the water quality index using eleven vital parameters. The WQI estimation was performed using the class 1 drinking water quality standards recommended by the South Africa Bureau of Standards (SABS) and the weighted arithmetic index (Table 2). The quality rating, *Q*_*n*_, was determined using Eq. 11:

11 $${Q}_{n}=\frac{{V}_{n}-{V}_{0}}{{S}_{n}-{V}_{0} }\times 100$$

where *Q*_*n*_ is the estimated rating for the *n*^th^ water quality, *V*_*n*_ is the average concentration of the positional parameter per sampling period and station, *S*_*n*_ is the common value provided by the class 1 SABS, and *V*_0_ is the ideal value (the constant value, 0, was applied for all other parameters except the parameter pH which is 7.0). WQI is obtained from the summation of the unit weight, *W*_*n*_, and the *Q*_*n*_ according to Eqs. 12 and 13:

12 $${WQI= \sum \nolimits_{i=0}^{n}{Q}_{n}\times W}_{n}$$

13 $${{W}_{n}= K/S}_{n}$$

where *K* is the constant for proportionality, deduced as the inverse of the summation of the Sn reciprocals as given by Eq. 14:

**Table 2:** The class 0 and 1 water quality standards and their unit weights for drinking provided by the South African Bureau of Standards

S/n	Parameter	Class 0	W_n0_	Class 1	W_n1_
1	EC	700	0.001	1500	0.001
2	pH	9	0.080	9.5	0.112
3	TDS	450	0.002	1000	0.001
4	Na	100	0.007	200	0.005
5	Mg	30	0.024	70	0.015
6	Ca	32	0.022	32	0.033
7	F	1	0.719	1.5	0.708
8	Cl	100	0.007	200	0.005
9	NO_3_ + NO_2_	6	0.120	10	0.106
10	SO_4_	200	0.004	400	0.003
11	K	50	0.014	100	0.011

14 $$K=1/\sum \nolimits_{i=0}^{n}{1/S}_{n}$$

According to the pioneer of WQI, Hortons (1965), WQI levels are categorized into excellent (WQI ≤ 25), good (26 ≤ WQI ≤ 50), poor (51 ≤ WQI ≤ 75), very poor (76 ≤ WQI ≤ 100), and unsuitable (WQI > 100). The chemometric analyses are performed in Microsoft Excel v.365. The index values generated per sampling period and point are prepared for time series analysis and class-based comparative analysis, using sequential Mann–Kendall trend analysis and confusion matrices, to visualize the temporal progradation of the series.

The temporal variation in water quality attributes across the selected hydrometric stations of BCH is statistically summarized in Table 3. The sodium adsorption ratio and nitrate samples for all the stations belong to the excellent category and class 0 of SABS (Table 2).

**Table 3:** The statistical description of the physicochemical parameters and the selected water quality indices

Stations	Statistics	TDS	Nitrate	SAR	RSI	LI	EC	ESP	WQI
	Mean	59.3	0.1	0.7	12.0	1.2	95.6	47.9	10.4
Upstream	Min	27.0	0.0	0.0	7.0	0.3	47.0	3.4	0.7
(*N* = 597)	Max	796.0	1.1	5.2	15.5	9.4	1209.0	71.9	70.7
	CV (%)	69.2	97.5	40.4	8.1	67.3	63.2	13.3	71.9
	Mean	249.9	0.2	1.5	9.1	1.2	396.0	50.6	19.2
Quencwe	Min	40.0	0.0	0.1	7.0	0.3	73.0	6.9	1.8
(*N* = 488)	Max	798.0	2.0	5.4	13.5	11.5	1196.0	73.2	52.6
	CV (%)	44.4	110.8	28.2	12.2	65.8	43.1	8.8	40.5
	Mean	651.1	0.3	4.7	7.8	1.6	1008.5	71.6	37.5
Ngqokweni	Min	50.0	0.0	0.7	6.4	0.7	85.4	43.9	5.8
(*N* = 429)	Max	1400.0	5.3	9.0	12.7	10.9	2150.0	83.8	74.2
	CV (%)	42.8	127.7	32.3	12.7	42.9	44.6	7.0	31.0
	Mean	720.4	0.9	4.4	7.4	1.9	112.9	66.9	42.0
Yellowwoods	Min	104.0	0.0	0.6	5.7	0.3	14.3	18.8	6.9
(*N* = 610)	Max	1803.0	8.0	10.1	12.2	6.6	288.0	89.5	317.7
	CV (%)	40.7	116.7	31.9	15.1	29.4	42.1	6.5	40.5

In contrast, ESP is permissible for Upstream (89%) and Quencwe (98%) and doubtful for Ngqokweni (96%) and Yellowwoods (97%) basins. TDS concentration varies from class 0 at the Upstream (100%) and Quencwe (99%) to class 0, 1, and 2 at Ngqokweni (26%, 61%, 13%) and Yellowwoods (20%, 66%, 14%). Similarly, EC varies from class 0 at the Upstream (100%) and Quencwe (95%) to class 0, 1, and 2 at Ngqokweni (28%, 55%, 17%) and Yellowwoods (21%, 58%, 21%) stations. Metal corrosion tendency due to chloride and sulfate concentration, according to LI, showed graduation for moderate and robust corrosion across the river course at Upstream (42% and 36%), Quencwe (66% and 28%), Ngqokweni (7% and 92%), and Yellowwoods (4% and 95%) basins. RSI varies enormously from the Upstream (100% highly corrosive), Quencwe (4% mild, 45% strongly, and 51% highly corrosive), Ngqokweni (17% encrusting, 27% mild, 45% strongly, and 11% highly corrosive), and Yellowwoods (5% strongly encrusting, 41% encrusting, 19% mild, 27% strongly, and 8% highly corrosive) stations. According to WQI, water quality is dominantly excellent at the Upstream (96%) and Quencwe (81%) and good at Ngqokweni (73%) and Yellowwoods (60%). Yellowwoods station exhibited poor (20%) and very poor (2%), and Ngqokweni station showed 14% poor water.

### Comparative analysis

Following the projection of EHE using SHDI, sequential Mann–Kendall (SMK) analysis was employed to project the temporal progradation water quality time series. At the same time, CM and WC provide categorical and periodic correlations.

#### Sequential Mann–Kendall analysis

Sequential Mann–Kendall analysis depicts the change in time value as an oscillation that either exhibits a positive or negative trend across the entire statistical sequence (Sneyers 1990; Owolabi et al. 2022).

The progradation series is projected by computing ranked value *y*_*i*_ (*i* = 1, 2, 3,…, *n*) from the original values in the series (*x*_1_, *x*_2_, *x*_3_,….,*x*_*n*_) and comparing its magnitude with *y*_*j*_, precedent stepwise value of *y*_*i*_, that is, *y*_*j*_ = *y*_*i*−1_ (where *j* = 1, 2, 3,…, *i* − 1). For every stepwise comparison, *n*_*i*_ is assigned to counts of *y*_*i*_ > *y*_*j*_, and its summation is calculated as statistic *t*_*i*_ to enable the computation of the mean distribution of the test statistics, *E(t*_*i*_*)*, and variance, *VAR*(*t*_*i*_), according to Eqs. 15–17:

15 $${t}_{i}= \sum \nolimits_{j=1}^{i}{n}_{i}$$

16 $${E(t}_{i})=\frac{i(i-1)}{4}$$

17 $${VAR(t}_{i})=\frac{i(i-1)(2i+5)}{72}$$

The deductions are parameterized for the computation of the prograde, *u*(*t*_*i*_), given by Eq. 18:

18 $${u(t}_{i})=\frac{[{t}_{i}-E\left({t}_{i}\right)]}{\sqrt{VAR({t}_{i})}}$$

The retrograde, *u*’ (*t*_*i*_), series are computed similarly from the end of the sequel to the first. The change point across the time series is obtained through the intercept produced by the prograde and retrograde series. The trend significance measures the extent of displacement from the confidence limit within the SMK graph. The daily streamflow data from 1972 to 2011 and some selected water quality parameters and indices which include TDS, EC, nitrates, SAR, EC, LI, RSI, and WQI were analyzed using sequential Mann–Kendall analyst and the Trend Change package version 1.2 prepared by Sneyers (1990) in the RStudio.

#### Confusion matrices

SHDI deductions were ranked; hence, the reference variables were classified according to the relative number of drought levels. The drought levels were grouped in the rows corresponding to the value intensity in the *i*^th^ row. In contrast, the classified reference variables were grouped in the columns relative to the value class in the *j*^th^ column. Based on the matrices, the predomination of drought intensity on the reference variable enables the generation of accurate prediction, assembled along the diagonal, *X*_*ii*_, the drought class, and the predicted model in class *i* and class *j*, respectively. The overall accuracy, OA, which provides the proportion of correct predicted classes of the comparative assessment, is given by Eq. 19:

19 $$OA=\frac{\sum_{i=1}^{l}{X}_{ii}}{N}$$

where *l* denotes the number of rows in the error matrix corresponding to the drought levels. *N* is the total number of reference variables. The model is said to be ideal when OA is 100%. The Kappa coefficient is obtained through Eq. 20:

20 $$K=\frac{N(\sum_{i=1}^{l}{X}_{ii})-(\sum_{i=1}^{l}{(x}_{i+}\times {x}_{+j}))}{{N}^{2}-(\sum_{i=1}^{l}{(x}_{i+}\times {x}_{+j}))}$$

where *x*_*i*+_ and *x*_+*j*_ are the pixels in row *i* and column *j*. Considering that the assessment was focused on the relativity of the water quality parameters on hydrological drought levels, the study dwelled on the sensitivity of water quality parameters to hydrological drought. The sensitivity assessment was computed using Eq. 21 (Altman and Bland 1994):

21 $$SNA=\frac{N(\sum_{i=1}^{l}{X}_{ii})}{(\sum_{i=1}^{l}{x}_{i+})}$$

This study manually co-matches the monthly SDHI to the monthly parameterized and indexed water quality to adopt the CM. Three scenarios of hydrological levels were maintained: 2-phase level comprising of the normal (*z* ≥  − 1) and dry (*z* <  − 1) spells; 3-phase level comprising of the wet (*z* > 1), normal (1.0 ≤ *z* <  − 1.0), and dry (*z* <  − 1); and the 7-phase levels described under the SPI approach. The water quality variables were classified using percentiles such that 0.5 (2-phase), 0.33 (3-phase), and 0.14 (7-phase) percentiles were maintained for the co-movement of water quality series to match the SHDI level per phase. The series was ranked in Microsoft Excel, while the CM was performed in RStudio using classification and regression training packages prepared by Kuhn (2008).

#### Wavelet coherence analysis

Wavelet coherence deduces the degree of association, the compelling correlation, and the phase shift between two variables (Torrence and Compo 1998), in this case SHDI and WQ variables. The Morlet wavelet computation deduces the wavelet coefficients at the configured time–frequency scale. In this case, the Fourier period for wavelet decomposition was preset to 1 to 256 days. The wavelet coherence was run 100 times at an optimum iteration time using the Hanning window type with no pre-whitening. The resulting projection provides detailed information on the relationship between *x*(*t*) and *y*(*t*). A smoothened spectral version of the cross-wavelet transform and time–frequency regression property of the relationship between *x*(*t*) and *y*(*t*) are captured in the computation of wavelet coherence defined in Eq. (15):22where

(*u, s*)
wavelet squared coherence (  ).
*S*: smoothing operator over time (*t*) and the scale of

The phase difference in the time series is computed to project the co-movement and phase relationship between the *x*(*t*) and *y*(*t*) according to Eq. 16:23where *Ḯ* and *Ṝ* denote the imaginary and real parts of the smoothed cross-wavelet transform, respectively.

24 $${W}_{n}^{xy}\left(u, s\right)= {W}_{n}^{x}\left(u, s\right)*{W}_{n}^{y}(u, s)$$

where *u* connotes the location, and *W*_*n*_^*x*^ and *W*_*n*_^*y*^ represent the individual integral continuous wavelet transform, defined as in Eq. 13 (Torrence and Compo 1998):

25 $${W}_{n}^{x}\left(u,s\right)=\sqrt{\frac{\delta t}{4s}}\sum \nolimits_{t=0}^{T-1}x\left(t\right){\Psi }_{0}^{*}*{\Psi }_{0}\left(\delta t\frac{\left(t-u\right)}{4s}\right)$$

This was based on the computation of the Morlet wavelet, as defined in Eq. 14:

26 $${\Psi }_{0}(t)={\pi }^{\frac{-1}{4}}{e}^{-i{\omega }_{0}t}{e}^{\frac{-{t}^{2}}{2}}$$

where *i* denotes the imaginary unit and ω_*0*_ the nondimensional frequency.

Black arrows show the phase relationship in the wavelet coherence plot at intense power correspondence. The direction of the black arrows describes the type of phase existing in the wavelet analysis. Arrows that plot eastward (westward) denote in-phase and positively correlated (out-phase and negatively correlated) time series. Also, arrows that plot northward (southward) reveal that the preceding time series leads (lags) the succeeding series by π/*2*. The wavelet analysis explains the time series’ current, short-, medium-, long-term correlation, and phase attributes. The projection presents the frequency in days along the vertical axis, while the temporal changes along the SHDI and WQ are shown on the horizontal axis. A contour line, computed through Monte Carlo simulation, is generated around the intense power spectrum. This indicates the zones of significance at the 5% level. A solid bell-shaped curved padding is generated to demarcate the possible erroneous zone at the beginning and end of the finite-length time series affected by the edge effect (Torrence and Compo 1998). The computation is performed in R studio.

### Corroboration of the water quality climate sensitivity investigation

The comparative assessment was corroborated by a spatio-temporal evaluation of land use/land cover changes (LULC), water quality (WQ) evolution, and ground-truthing. The 72-class land use/land cover (LULC) maps of South Africa for 1990 and 2020 were downloaded from the Department of Environment and Agriculture website to project the recent changes across the BCH in thirty years. The water-related, forest-related, cultivation-plantations, residents, bushes, grasslands, mines, schools, and bare portions are regrouped into seven land cover types: waterbodies, forest, shrubland, farmland, grassland, built-up area, and bare ground. The percentage rate of change was calculated using the cell counts fraction. The study proposed a simplified approach for the calculation of landscape categories from the featured LULC types based on the propensity of human use;

27 $$\mathrm{Natural\;landscape}=\mathrm{ Forest}+\mathrm{Waterbodies}+\mathrm{Shrubland}$$

28 $$\mathrm{Semi}\_\mathrm{natural\;landscape}=\mathrm{\;Farmland}+(\frac{\mathrm{Grassland}}{2})$$

29 $$\mathrm{Artificial\;landscape}=\mathrm{ Built}\_\mathrm{up}+\mathrm{Bareground}+(\frac{\mathrm{Grassland}}{2})$$

where the values of the LULC types were drawn from their percentage ratio.

**Fig. 2:**
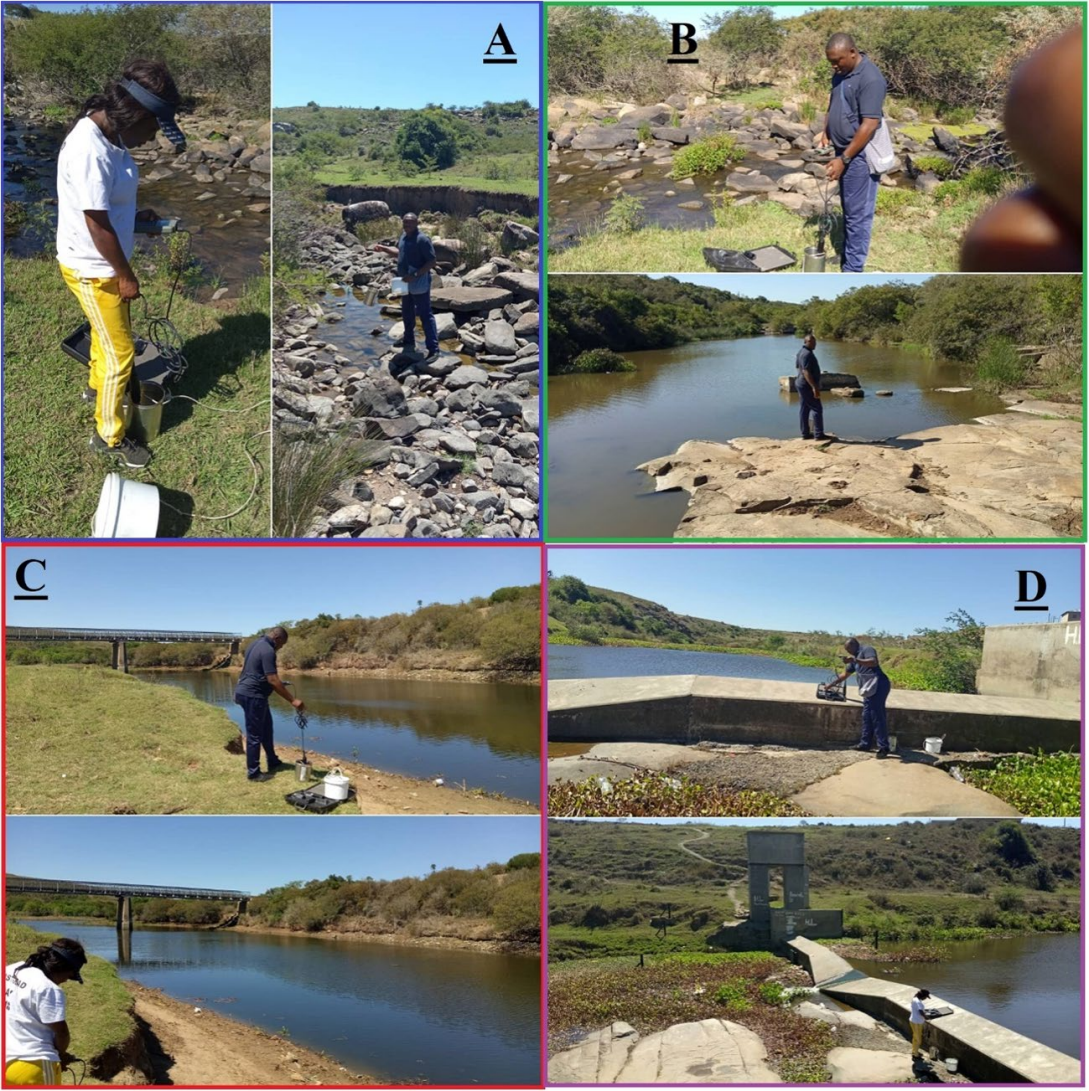
Picture of the ground-truthing exercise and the in situ assessment of BCH physicochemical parameters, at the hydrometric stations of **A** Upstream, **B** Quencwe, **C** Ngqokweni, and **D** Yellowwoods rivers

Ground-truthing was conducted to strengthen the landscape assessment and to collect surface water samples for physicochemical parameter assessment at the hydrometric stations. Surface water samples were collected for in situ data collection and experimental purposes in triplicate on 06^th^ February 2019, using a 50-ML GL 32 cap bottle (Fig. 2). The sampling plastic bottles were carefully rinsed using distilled water before sampling to remove and neutralize any impurity present earlier. All samples were collected a few meters away from the river’s edges. TDS data, alongside other physicochemical parameters, were recorded at the site using portable meter Hach HQ40D and DR900 Multiparameter Portable Colorimeter. Water quality assessment for total sodium, calcium, chloride, and bicarbonate was experimented with using the atomic absorption spectrometry approach in the water chemistry laboratory of the Department of Chemistry, University of Fort Hare, Eastern Cape, South Africa. The mean annual concentration of TDS, sodium, calcium, chloride, and bicarbonate for 1977, 1981, 1986, 1995, 2000, 2005, and 2011 were collated to perform the water quality evolution assessment together with the one obtained in the field using a Gibbs diagram.

Gibbs diagram provides the inference for the vital environmental processes responsible for the water quality evolution mechanism based on the refreshing, evapoconcentration, mineralization, mixing, and resident timing of water within its reservoir (Gibbs 1970). Gibbs diagram was obtained through the scattered plot of total dissolved solids against sodium/(sodium + calcium) ratios for the cations and chloride/(chloride + bicarbonate) for the anions (Marandi and Shand 2018).

## Results

### Standard hydrological drought index of Buffalo catchment headwater

The comparative evaluation of the SHDI-3 and SHDI-12 graphs (1971–2012) of the four sub-catchments is shown in Fig. 3. SHDI-12 charts provide a better drought intensity projection than SHDI-3 graphs characterized by numerous insignificant nodes of moderate droughts.

**Fig. 3:**
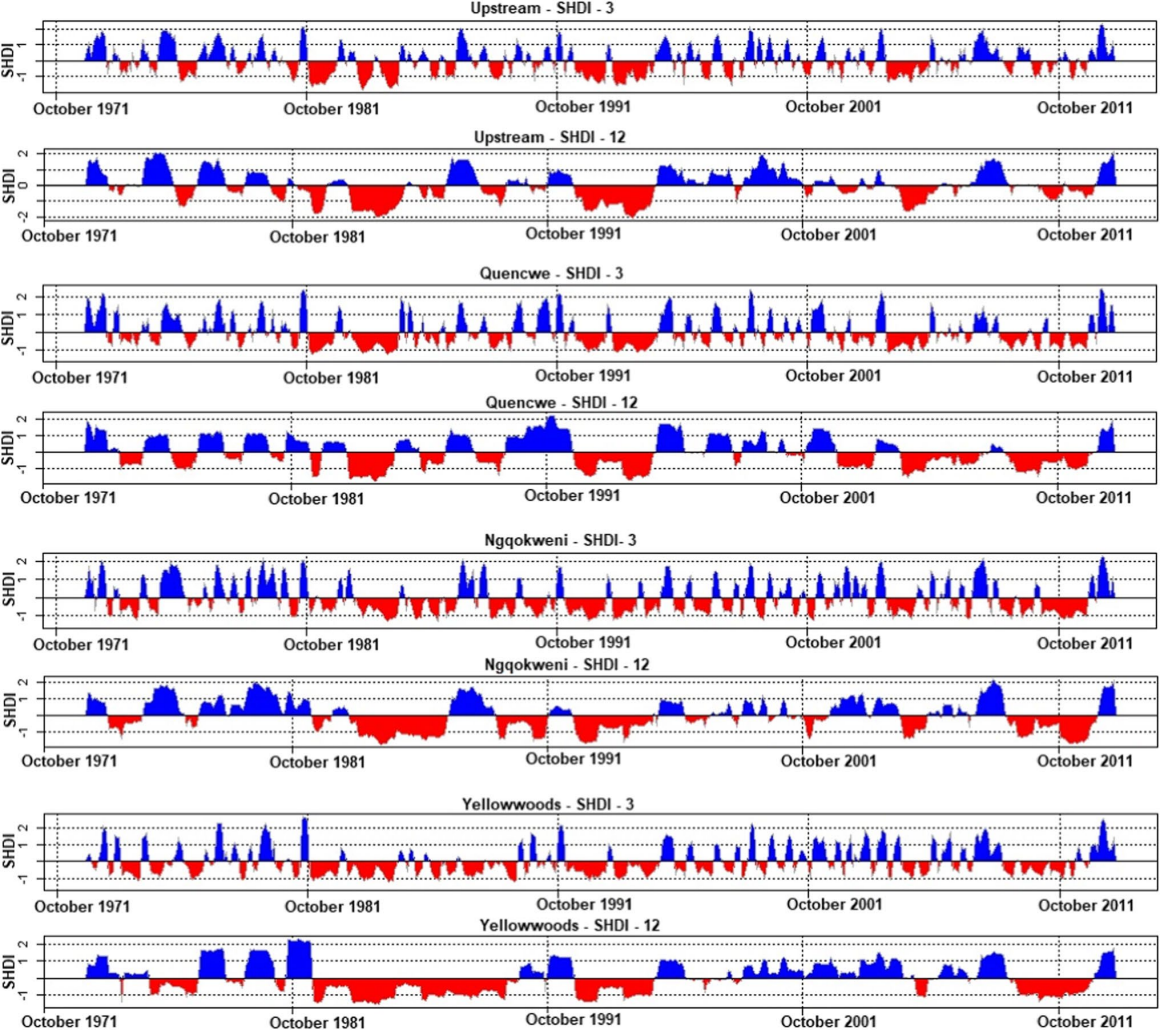
The standard hydrological drought index graphs of selected hydrometric gauging stations of Buffalo catchment headwater (October 1971 to March 2012)

The Upstream basin showed the highest drought severity in the second decade, while the Quencwe basin depicts the longest drought severity (17 years) with a prolonged duration from 2002 to 2012. Ngqokweni basin exhibited the highest number of SHDI-3 moderate drought amplitudes, 23 nodes compressed into 10 nodes in SHDI-12, compared to Upstream (20 > 6 nodes), Yellowwoods (11 to 11 nodes), and Quencwe (8 > 6 nodes) basins. The year 1982 to 1984 exhibited the most vigorous drought severity across the four basins, with variations in the periodicity of drought across the basins. Quencwe basin also showed the most recurrent extreme wetness (1981, 1991, 1999, and 2012), followed by Yellowwoods (1981 and 2012) basin.

The assessment showed a remarkable spatial variation in river quantitative response to the extreme hydrological events across the temporal gap. The uphill rivers exhibit tangible extreme wetness recurrence and consequential low drought exceedance in the first decade compared to the fourth decade, which showed a significant increase in drought intensity, thus suggesting the possibility of a considerable streamflow decline. Contrarily, the foothill rivers depict a tangible recession with severe droughts in the second decade, which resurged in the third and are characterized by significant high wetness duration in the fourth decade, thus, insinuating a significant river resurgence in the fourth decade.

### Water quality trend assessment

**Fig. 4:**
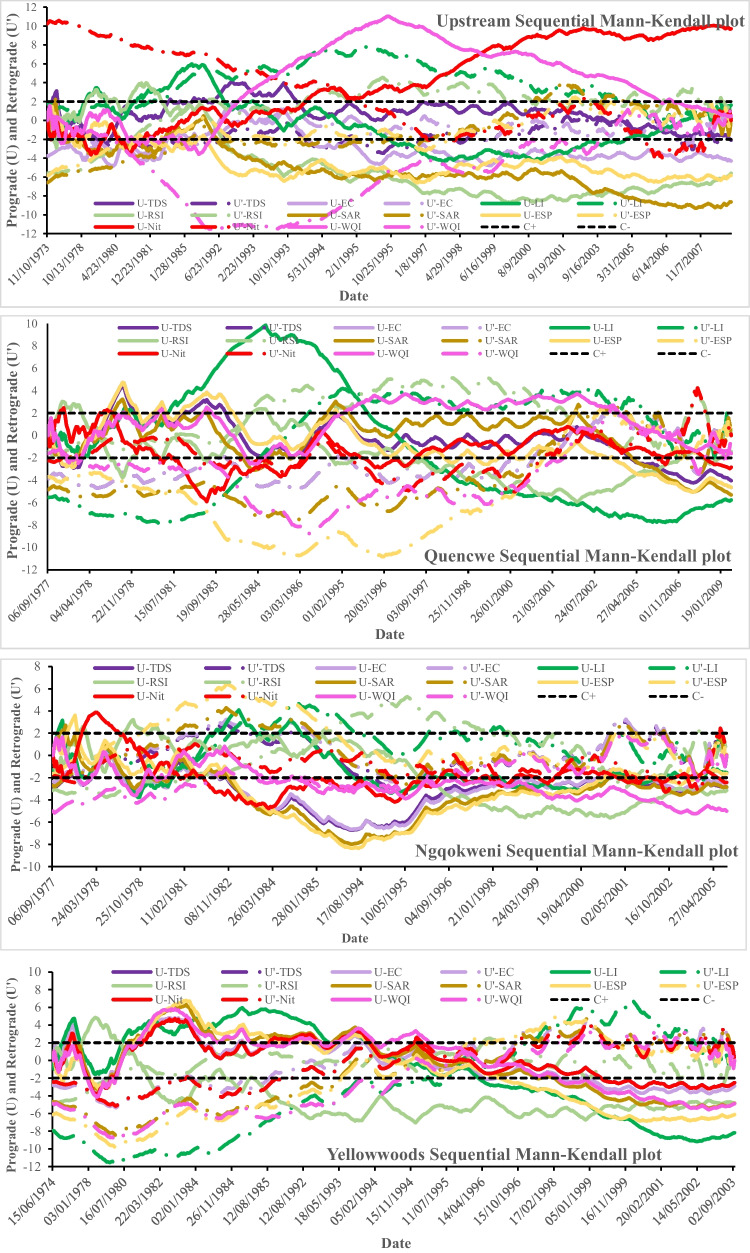
The sequential Mann–Kendall plots of selected physicochemical parameter series (TDS, total dissolved solids; EC, electrical conductivity; LI, Larson index; RSI, Ryznar stability index; SAR, sodium adsorption ratio; ESP, exchangeable sodium percentage; Nit, nitrate; WQI, water quality index) of October 1971 to March 2012 for Buffalo Upstream (first), Quencwe (second), Ngqokweni (third), and Yellowwoods (fourth) basins

The sequential Mann–Kendall analysis of the water quality parameters was evaluated with no pre-whitening to preserve the originality of the data trend and its outliers at the 95% confidence limit (Fig. 4). All the WQ parameters except nitrate and WQI exhibit significant negative trends in the four hydrometric stations. Nitrate depicted positive trends at Upstream and Yellowwoods stations, while WQI depicted positive trends at Upper Course stations (Upstream and Ngqokweni). By comparing the periods of abrupt hydrological change from intense wetness to severe drought at the Upstream (1971–1986; 1995–2001), Quencwe (1981–1983; 1990–1992), Ngqokweni (1985–1991; 1995–2001), and Yellowwoods (1977–1982), water quality parameters were significantly altered with varying sensitivity (Fig. 4). However, a critical inspection of the period of extreme dryness based on the SDHI-3 and -12 showed that only RSI exhibits a continuously decreasing trend, while LI, SAR, and WQI exhibited intermittent increases in trend. In Quencwe, Ngqokweni, and Yellowwoods stations, all the WQ variables depicted decreasing trends with pronounced intermittency on nitrate unlike LI and RSI, which showed increasing trends. The sequential Mann–Kendall change point (point of interception of the prograde and the retrograde) suggesting the abrupt response to environmental changes varies decreasingly from the upper to the lower course stations. Only Upstream showed haphazard WQ change points (17) with 1980 and 1981 being the most consistent years across the variables. Quencwe, Ngqokweni, and Yellowwoods showed 17, 3, and 1 consistent change points across the WQ variables’ timeframe, respectively. Only Yellowwoods depicted 1994, the period that marked the end of significant drought in the basin. Ngqokweni flagged 1977–1978, the period of abrupt decline in extreme wetness, and Quencwe identified 1978, 1985 (the period of abrupt break in drought), and other extreme dryness period, ending with 2007, the post-drought period.

### Comparative analysis based on categorical correlation

The categorical interrelationship between drought levels and water quality divisions was examined using confusion matrices at a *p*-level < 0.05 (Table 4). The result shows a decline in the overall accuracy and Kappa coefficient with increased drought level segregation, indicating the eventful significance of lumping the drought levels when evaluating water quality alteration. According to the 2-phase, wetness and dryness seasonality are moderately correlated to water quality alteration based on the overall accuracy (0.43 to 0.73). The deductions of the 3- and 5-phase deductions reveal that wetness, flow normalcy, dryness, and their further division are equally important for water quality response weightage (based on the SA) despite the low overall accuracy (0.22–0.45 and 0.11–0.23) and low representation by the Kappa coefficient (0–0.17 and 0–0.05). By substantiating the overall accuracy and Kappa coefficient scores in 2-phase, CM projected EC (Upstream), TDS, WQI (Quencwe), and SAR (Ngqokweni and Yellowwoods) as the peculiar parameters.

**Table 4:** The confusion matrices of drought levels with water qualities for the selected hydrometric stations in Buffalo River (*OA* overall accuracy, *KC* Kappa coefficient, *SA* sensitivity analysis)

Stations	WQ	2-Phase			3-phase			5-phase		
		OA	KC	SA	OA	KC	SA	OA	KC	SA
Upstream	TDS	0.56	0.13	0.86	0.40	0.08	0.66	0.21	0.05	0.67
	Nitrate	0.73	* − 0.01*	0.79	0.27	* − 0.11*	0.61	0.16	* − 0.01*	0.70
	SAR	0.52	0.08	0.84	0.35	*0.00*	0.58	0.16	* − 0.02*	0.56
	RSI	0.48	* − 0.04*	0.77	0.31	* − 0.03*	0.63	0.16	*0.00*	0.66
	LI	0.43	* − 0.14*	0.72	0.32	* − 0.02*	0.66	0.17	0.01	0.73
	EC	0.57	0.14	0.86	0.37	0.05	0.62	0.19	0.02	0.59
	ESP	0.48	* − 0.03*	0.77	0.31	* − 0.04*	0.61	0.17	0.01	0.69
	WQI	0.51	0.03	0.80	0.36	0.04	0.65	0.15	* − 0.01*	0.56
Quencwe	TDS	0.57	0.14	0.96	0.40	0.09	0.74	0.20	*0.00*	0.66
	Nitrate	0.42	* − 0.15*	0.82	0.30	* − 0.06*	0.75	0.17	* − 0.03*	0.77
	SAR	0.56	0.12	0.96	0.38	0.07	0.76	0.21	0.01	0.66
	RSI	0.44	* − 0.12*	0.84	0.31	* − 0.04*	0.78	0.16	* − 0.05*	0.68
	LI	0.50	0.01	0.90	0.32	* − 0.02*	0.73	0.18	* − 0.02*	0.66
	EC	0.56	0.12	0.96	0.41	0.11	0.77	0.22	0.02	0.68
	ESP	0.48	* − 0.05*	0.87	0.33	*0.00*	0.77	0.18	* − 0.02*	0.73
	WQI	0.57	0.14	0.96	0.40	0.09	0.76	0.22	0.03	0.66
Ngqokweni	TDS	0.49	*0.02*	0.86	0.38	0.08	0.70	0.22	0.02	0.72
	Nitrate	0.43	* − 0.13*	0.8	0.22	* − 0.08*	0.51	0.18	* − 0.02*	0.6
	SAR	0.53	0.06	0.90	0.32	0.06	0.77	0.19	* − 0.01*	0.66
	RSI	0.46	* − 0.07*	0.83	0.22	* − 0.08*	0.57	0.11	* − 0.05*	0.63
	LI	0.53	0.05	0.90	0.28	*0.00*	0.57	0.22	0.02	0.60
	EC	0.49	0.02	0.86	0.31	0.05	0.77	0.22	0.02	0.70
	ESP	0.53	* − 0.06*	0.90	0.27	* − 0.01*	0.63	0.23	0.04	0.65
	WQI	0.50	*0.00*	0.87	0.31	0.05	0.69	0.21	0.01	0.67
Yellowwoods	TDS	0.53	0.06	0.98	0.41	0.11	0.76	0.20	*0.00*	0.73
	Nitrate	0.47	* − 0.06*	0.92	0.33	* − 0.01*	0.72	0.17	* − 0.03*	0.64
	SAR	0.55	0.10	1.00	0.42	0.14	0.78	0.20	0.01	0.68
	RSI	0.48	* − 0.05*	0.93	0.35	0.02	0.82	0.19	* − 0.01*	0.84
	LI	0.52	0.05	0.52	0.39	0.09	0.72	0.20	0.00	0.71
	EC	0.54	0.08	0.99	0.45	0.17	0.82	0.19	* − 0.01*	0.70
	ESP	0.52	0.05	0.97	0.37	0.05	0.69	0.20	0.01	0.68
	WQI	0.54	0.08	0.99	0.41	0.11	0.76	0.21	0.02	0.68

The italicized numbers indicate the quotient of the anti-phase relationship between the water quality variable and the drought level

Kappa coefficient is generally low for all the drought-concentration correlations (0 to 0.17), thus suggesting the relevance of agent causation over event causation, which varies across the stations. Ranking from extreme wetness (least) to extreme drought (most), the consistency of the positive (negative) Kappa coefficient of TDS, SAR, EC, and WQI (nitrate, RSI, and ESP) suggests the astatic correspondence of the phase (antiphase) of the parameters in the direction of extreme hydrological events. Hence, TDS, SAR, EC, and WQI concentration increases as drought persists, while nitrate, RSI, and ESP concentrations are triggered by prolonged wetness. Notably, the Kappa coefficient obtained in the 3-phase (0–0.17) compared to the 2-phase (0–0.14) is higher for the water qualities of the watershed with artificial landscape characteristics (Yellowwoods and Ngqokweni).

Despite the weak project and relativity of drought events (based on KC and OA), the assessment depicts a high degree of sensitivity (3-phase, 5-phase). The evaluation reveals that extreme hydrological events can alter the water facie and its suitability for drinking, irrigation, and other industrial purposes and complicate the function of the ecological system. The highest level of sensitivity occurs at Yellowwoods (RSI), followed by Quencwe (Nitrate), Ngqokweni (TDS), and at the Upstream (LI), corresponding to the sequential Mann–Kendall intermittency.

Moreover, by applying the sensitivity threshold of ≥ 0.75, most indicators at Yellowwoods, Quencwe, and a few at Ngqokweni are flagged as sensitive to drought impact. The findings on the most sensitive indicators under the 5-phase correspond to the depth of water quality degradation captured by the sequential Mann–Kendall analysis. Overall, with the 5-phase drought level segregation characterized by reduced accuracy and representation, the confusion matrices share a similar deduction with the sequential Mann–Kendall plot, which showed a drastic disruption of concentration load in response to the impact of hydrologic extremes.

### Daily streamflow-concentration co-movement assessment

**Fig. 5:**
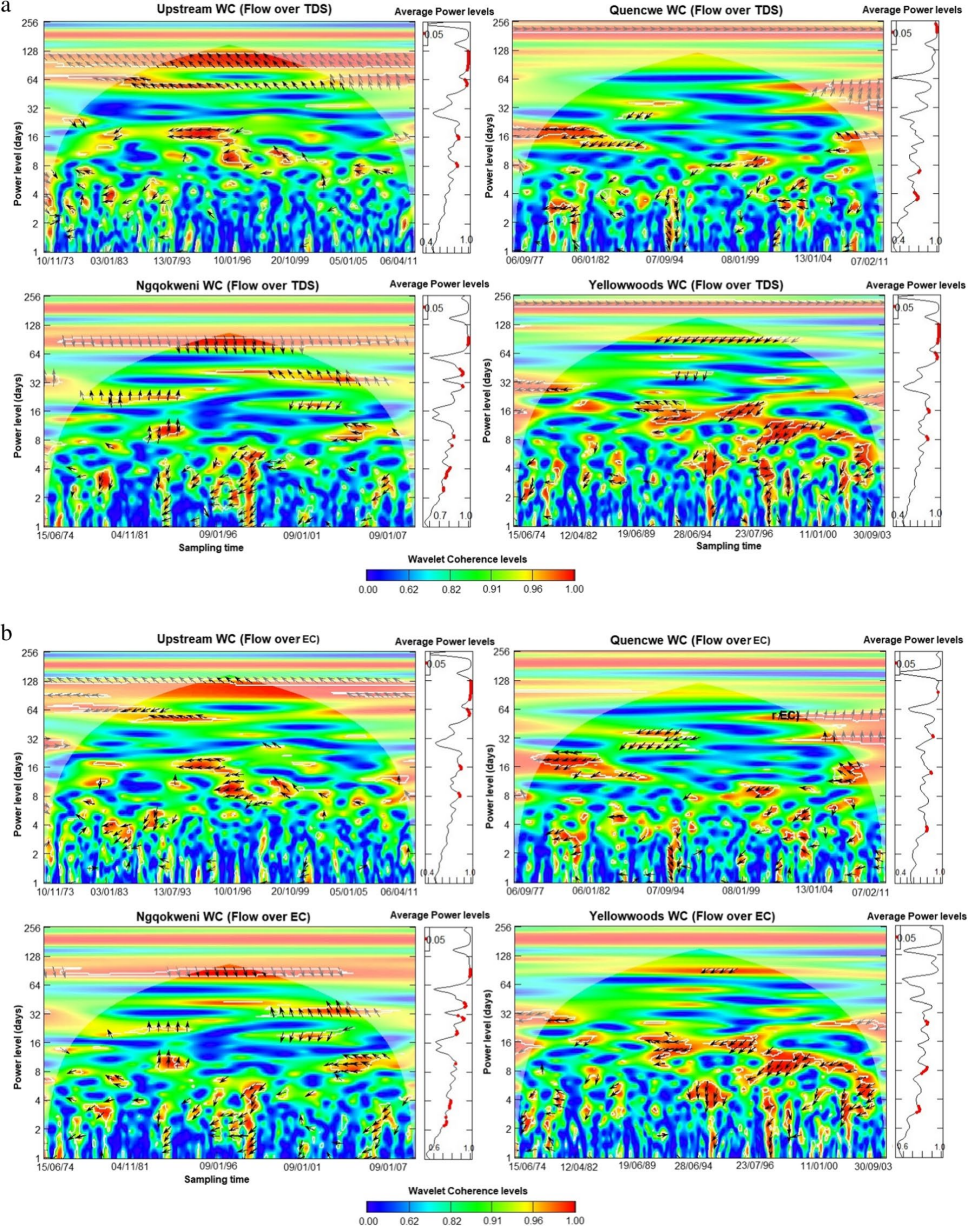

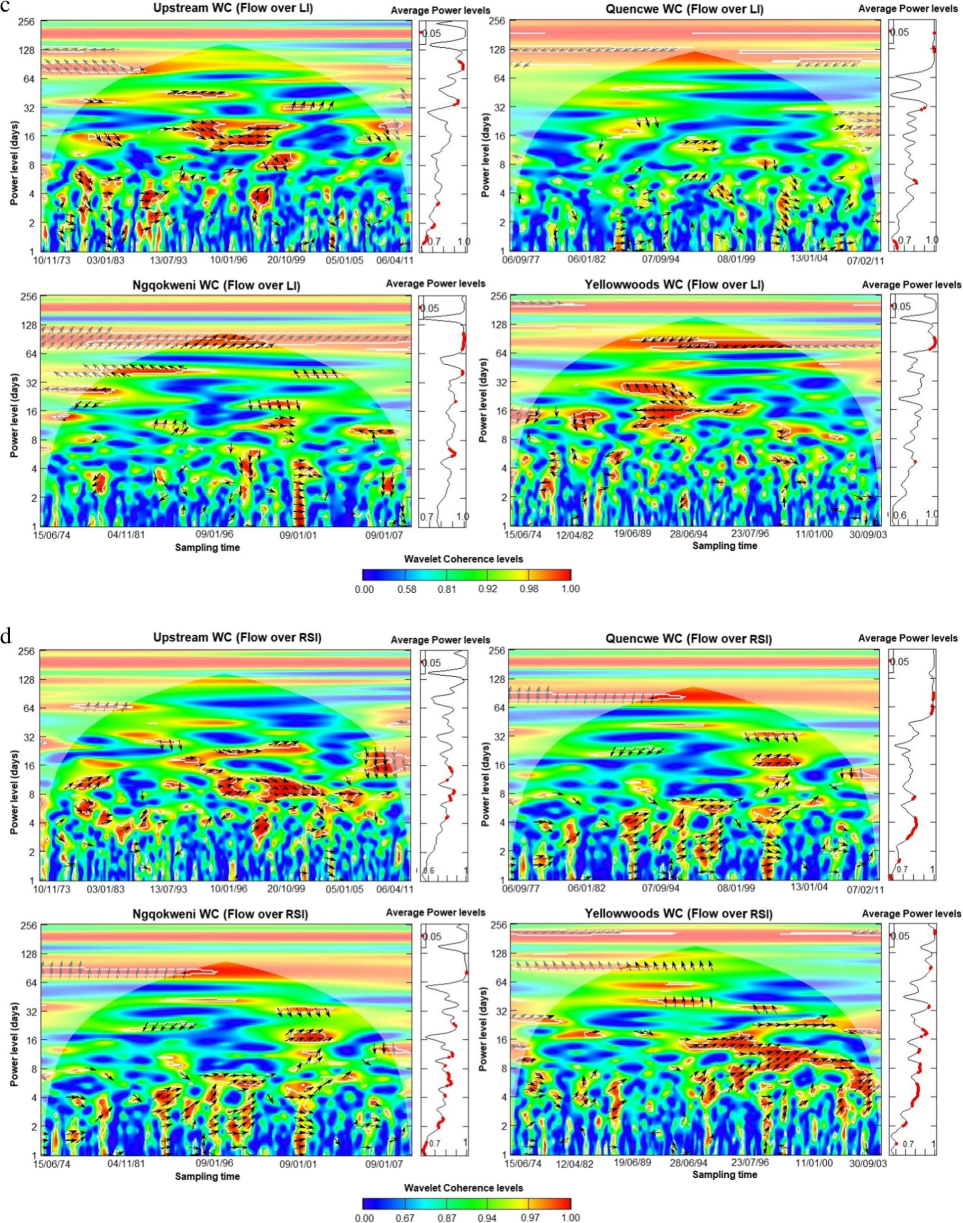

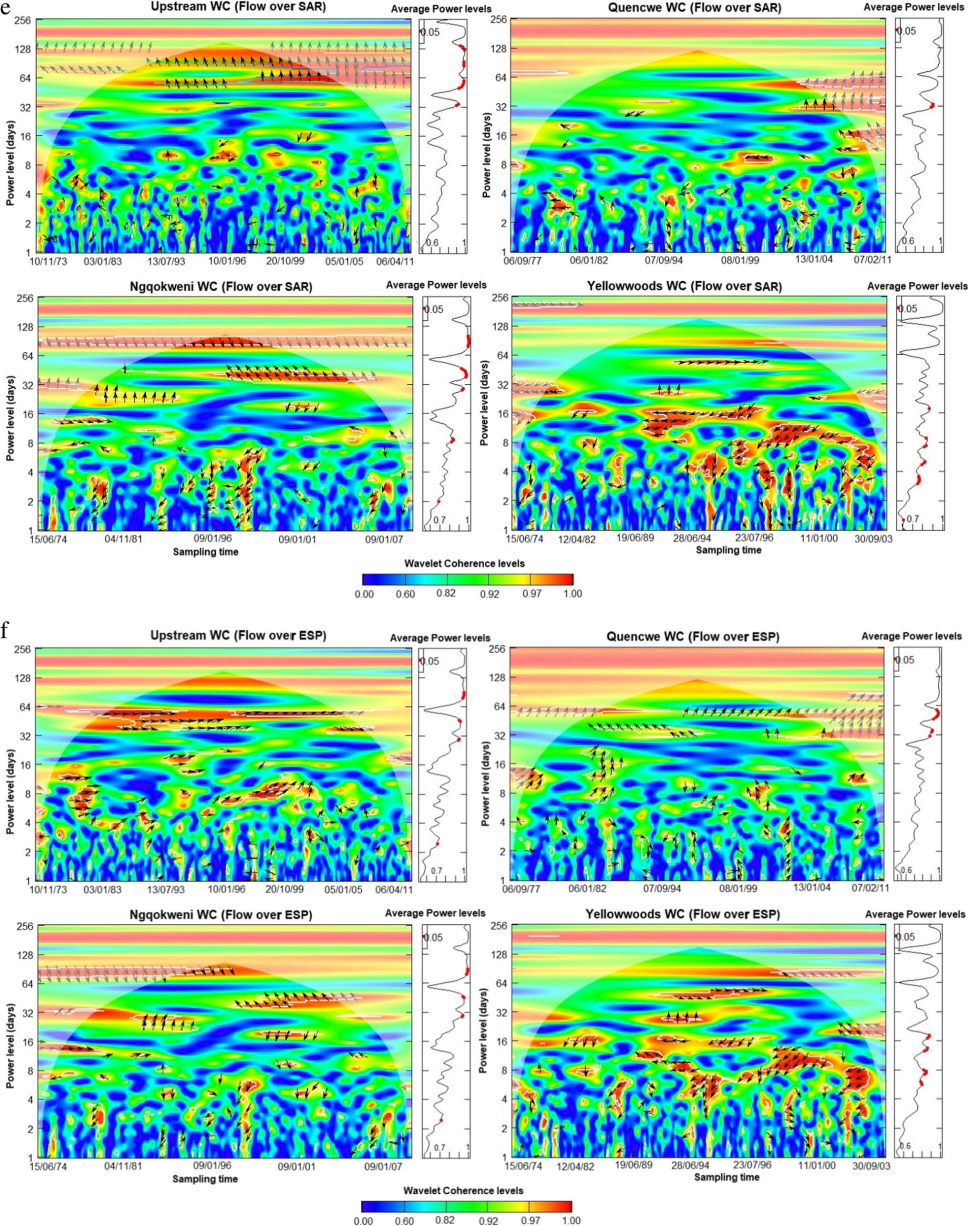

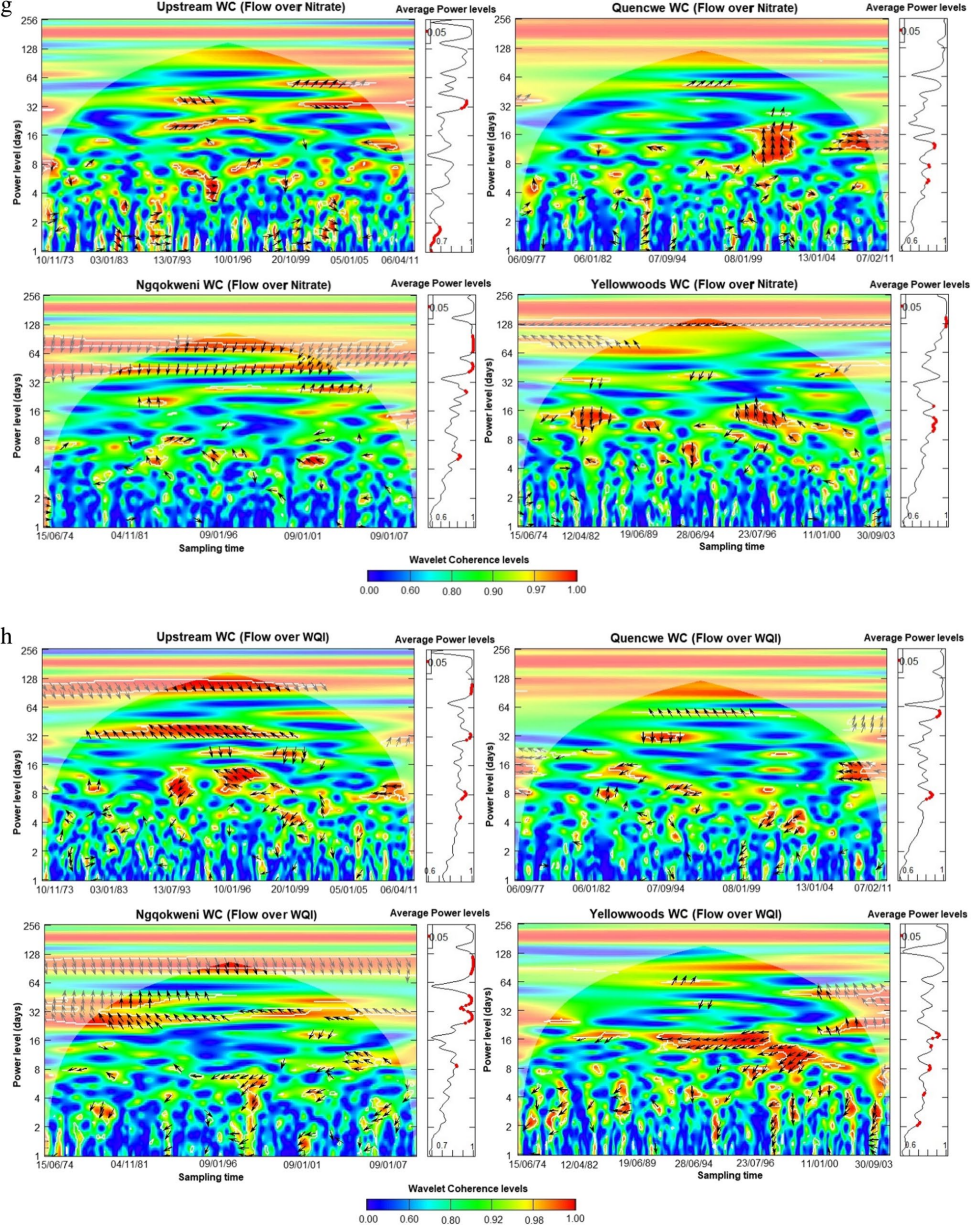
**a**. Wavelet coherence analysis for streamflow—total dissolved solids concentration for Buffalo hydrometric stations (October 1971 to March 2012). **b** Wavelet coherence analysis for streamflow—electrical conductivity for Buffalo hydrometric stations (October 1971 to March 2012). **c** Wavelet coherence analysis for streamflow—Larson index for Buffalo hydrometric stations (October 1971 to March 2012). **d** Wavelet coherence analysis for streamflow—Ryznar stability index for Buffalo hydrometric stations (October 1971 to March 2012). **e** Wavelet coherence analysis for streamflow—sodium adsorption ratio for Buffalo hydrometric stations (October 1971 to March 2012). **f** Wavelet coherence analysis for streamflow—exchangeable sodium percentage for Buffalo hydrometric stations (October 1971 to March 2012). **g** Wavelet coherence analysis for streamflow—nitrate concentration for Buffalo hydrometric stations (October 1971 to March 2012). **h** Wavelet coherence analysis for streamflow—water quality index for Buffalo hydrometric stations (October 1971 to March 2012)

The wavelet scalograms provide the relationship between the daily streamflow and co-sampled parameter component, plotted on the *y*-axis (Fig. 5 a–h). The assessment shows that the covariance between streamflow and water quality parameters is moderate to excellent (${R}_{n}^{2}\left(u,s\right)\ge 0.5$), especially within the spatio-temporal domain of 2 days to about 5 months periodicity based on the wavelet coherence level. Using the power level, the average coherence between the water quality parameters and the streamflow is significantly correlated at the mid-term (8 to 32-day) and long-term scales at the 0.05 significance level. All the water quality parameters exhibit an inverse co-movement with streamflow progression, except for LI and RSI, alluding to the findings of the confusion matrices and sequential Mann–Kendall trend contrariety.

Lead-lag co-movement varies depending on the variables underpinning the order of co-movement. For instance, streamflow leads over five WQ parameters and lags behind LI, nitrates, and WQI concentrations. Also, spatial variation exists in the length of the significant average power level. The upper course stations exhibit shorter periodicity for significant intermediate power levels than the lower course in almost all the variables, thus correlating the sequential Mann–Kendall change points. Moreover, some parameters showed significant long-term correlations compared to the lower course, typifying the significance of landscape quality.

Across all the stations, TDS sensitivity to streamflow was inverse except in the Upstream, which began after 8 days (Fig. 5 a). As a similitude to TDS, EC sensitivity extends to 64 days from 8 for Upstream and Ngqokweni, while it is shorter for Yellowwoods (24 days) and longer for Quencwe (128 days) (Fig. 5 b). LI sensitivity to streamflow is direct; that is, water propensity for metal corrosion due to sulfate and chloride concentration is triggered by increased wetness except in the Yellowwoods, where it is inverse, with significant scalar concentration occurring after 6 days (Fig. 5 c). In contrast to LI, RSI scalar sensitivity to streamflow varies for the four stations: Quencwe (1–8 days), Ngqokweni (1–16 days), Yellowwoods (2–20 days), and Upstream (4–16 days) (Fig. 5 d). RSI sensitivity compares with LI based on its direct relationship with wetness progradation except in Quencwe stations (Fig. 5 e). Most of the sensitivity of SAR to streamflow is captured with 32 months of prolonged drought, as captured by the inverse variation. While SAR sensitivity in the Upstream (32–128 days) and in Quencwe (32 days) was delayed for a month, in Ngqokweni (8 and 32 days) and Yellowwoods (3–32 days), SAR depicted an early response to streamflow. ESP co-movement with streamflow progression is complicated with a short-term inverse relationship and the alternation into direct covariance in the long term (Fig. 5 f). All the stations exhibit a direct ESP-streamflow co-movement in the long-term (64 days). However, Upstream (1–2 days) and Quencwe (2–4, 32 days), exhibited short-term inverse co-movement compared to Ngqokweni and Yellowwoods, which depict inverse co-movements at the short and mid-term periodicities.

### Corroboration of the study

#### Land use/land cover mapping

**Fig. 6:**
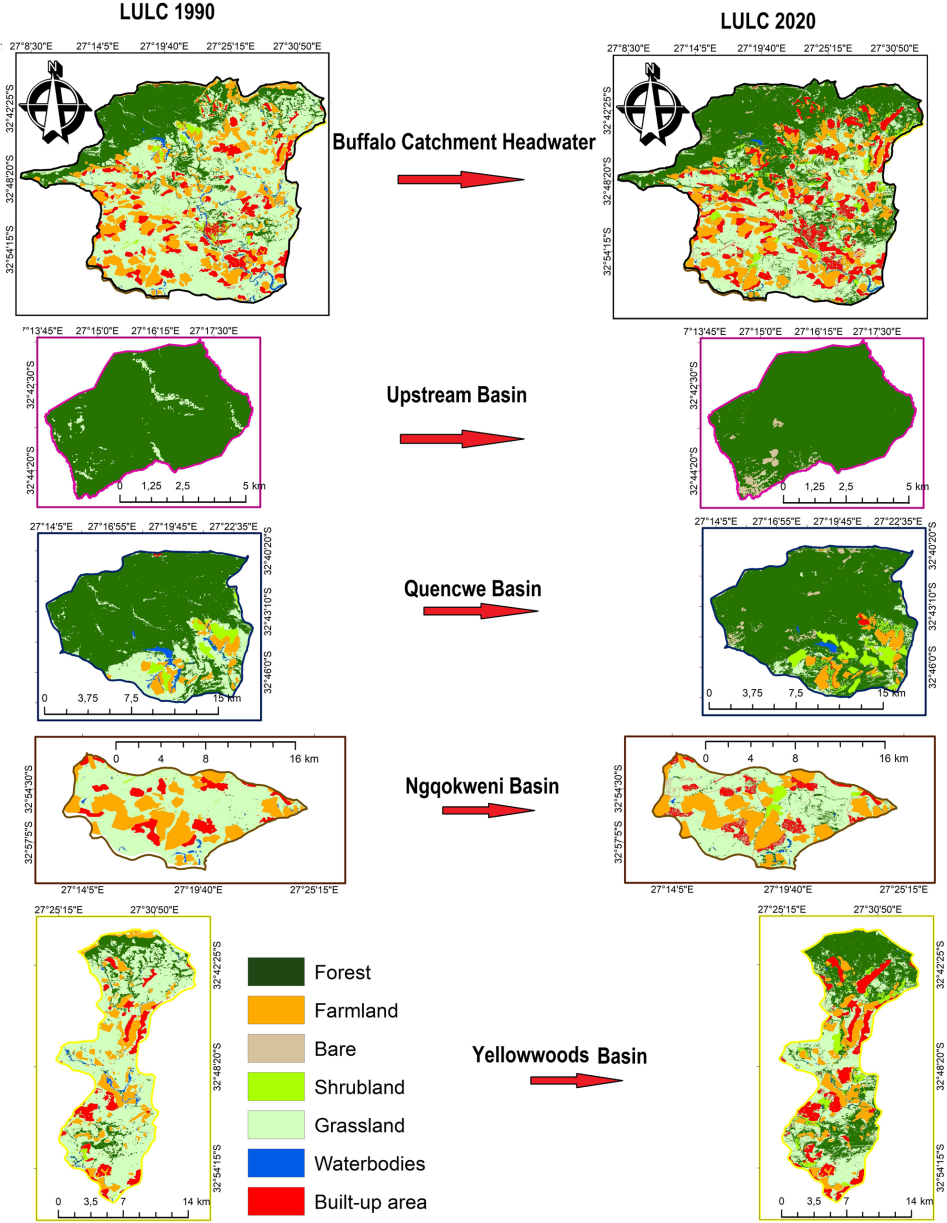
The land use /land cover change map of Buffalo catchment headwater shows the changes across Upstream, Quencwe, Ngqokweni, and Yellowwoods basins

A spatial analysis of the LULC change trajectory was undertaken to decipher how changes in the proportion of anthropogenic activities induce the water quality trend (Fig. 6; Tables 5 and 6).The result shows that the BCH is mainly rural, and economic activities in the area are most possibly agrarian due to the scanty built-up area and the proportion of vegetated cover to the developed regions. Notably, the LULC across all BCH basins exhibits a reduction in the waterbody cover and accedes to the possible severity of the hydrological drought in BCH, as depicted by the SHDI plots. Moreover, WQ concentrations reflect the LULC characters, as presented in Table 3.

**Table 5:** The land use/land cover changes over 30 years

	*LULC*	Cell counts		Ratio per basin (%)		Trajectory
	*Features*	*1990*	*2020*	*1990*	*2020*	*Rate*
Upstream	*Forest*	30,871	74,811	96.34	96.05	− 0.30
	*Grassland*	1157	383	3.61	0.49	− 86.38
	*Shrubland*	14	0	0.04	0.00	− 100.00
	*Bare*	2	2692	0.01	3.46	55,277.38
Quencwe	*Waterbodies*	2986	2591	1.68	0.61	− 63.61
	*Forest*	125,741	330,337	70.63	77.82	10.17
	*Grassland*	33,412	25,636	18.77	6.04	− 67.82
	*Shrubland*	6361	30,577	3.57	7.20	101.59
	*Farmland*	9284	21,908	5.22	5.16	− 1.04
	*Built-up area*	125	3044	0.07	0.72	921.24
	*Bare*	114	10,414	0.06	2.45	3730.93
Ngqokweni	*Waterbodies*	979	1707	0.76	0.54	− 29.02
	*Forest*	203	12,310	0.16	3.91	2368.41
	*Grassland*	77,504	167,043	60.48	53.06	− 12.27
	*Shrubland*	811	6927	0.63	2.20	247.68
	*Farmland*	35,706	88,901	27.86	28.24	1.35
	*Built-up area*	12,712	27,636	9.92	8.78	− 11.51
	*Bare*	238	10,304	0.19	3.27	1662.32
Yellowwoods	*Waterbodies*	2841	1919	1.22	0.34	− 71.84
	*Forest*	29,298	197,546	12.59	35.39	181.12
	*Grassland*	146,381	187,009	62.90	33.50	− 46.74
	*Shrubland*	1657	23,964	0.71	4.29	502.97
	*Farmland*	33,057	71,781	14.20	12.86	− 9.47
	*Built-up area*	18,697	73,143	8.03	13.10	63.10
	*Bare*	801	2846	0.34	0.51	48.14

**Table 6:** The designation of the LULC plots into landscape types

Basins	Year	Natural	Semi-natural	Artificial
*Upstream*	*1990*	96.4	1.8	1.8
	*2019*	96.1	0.2	3.7
*Quencwe*	*1990*	75.9	14.6	9.5
	*2019*	85.6	8.2	6.2
*Ngqokweni*	*1990*	1.6	58.1	40.3
	*2019*	6.7	54.8	38.6
*Yellowwoods*	*1990*	14.5	45.7	39.8
	*2019*	40.0	29.6	30.4

Despite the dispersivity of the farmland, the concentration of nitrate and SAR is within the South African Bureau of Standards recommended limit. At the same time, the ESP result is consistent with farmland proportions in the Yellowwoods and Ngqokweni basins. The TDS, EC, LI, RSI, and WQI concentrations correspond to the landscape attributes exhibited by the basins within the 30-year change (Table 6). Considering the natural landscaping of the Upstream and Quencwe basins and the contrasting diversity in the Ngqokweni and Yellowwoods basins (Table 6), the LULC map suggests that EHE majorly drives the existence of significant trends across the WQ indices based on the similarity of the curve and trend direction. Overall, no tangible change that can trigger elevated concentration of WQ is exhibited by the built-up and farmland cover across all the basins. Only the Quencwe basin demonstrates a substantial potential which is still inconsequential in the relative percentage ratio. LULC confirms that the approach engaged in assessing the relationship between EHE and WQ indices helps in normalizing the effect of outliers steered by non-point sources. Hence, the LULC confirms that BCH's water quality trend alteration is mainly driven by EHE influence.

#### Gibbs plot

**Fig. 7:**
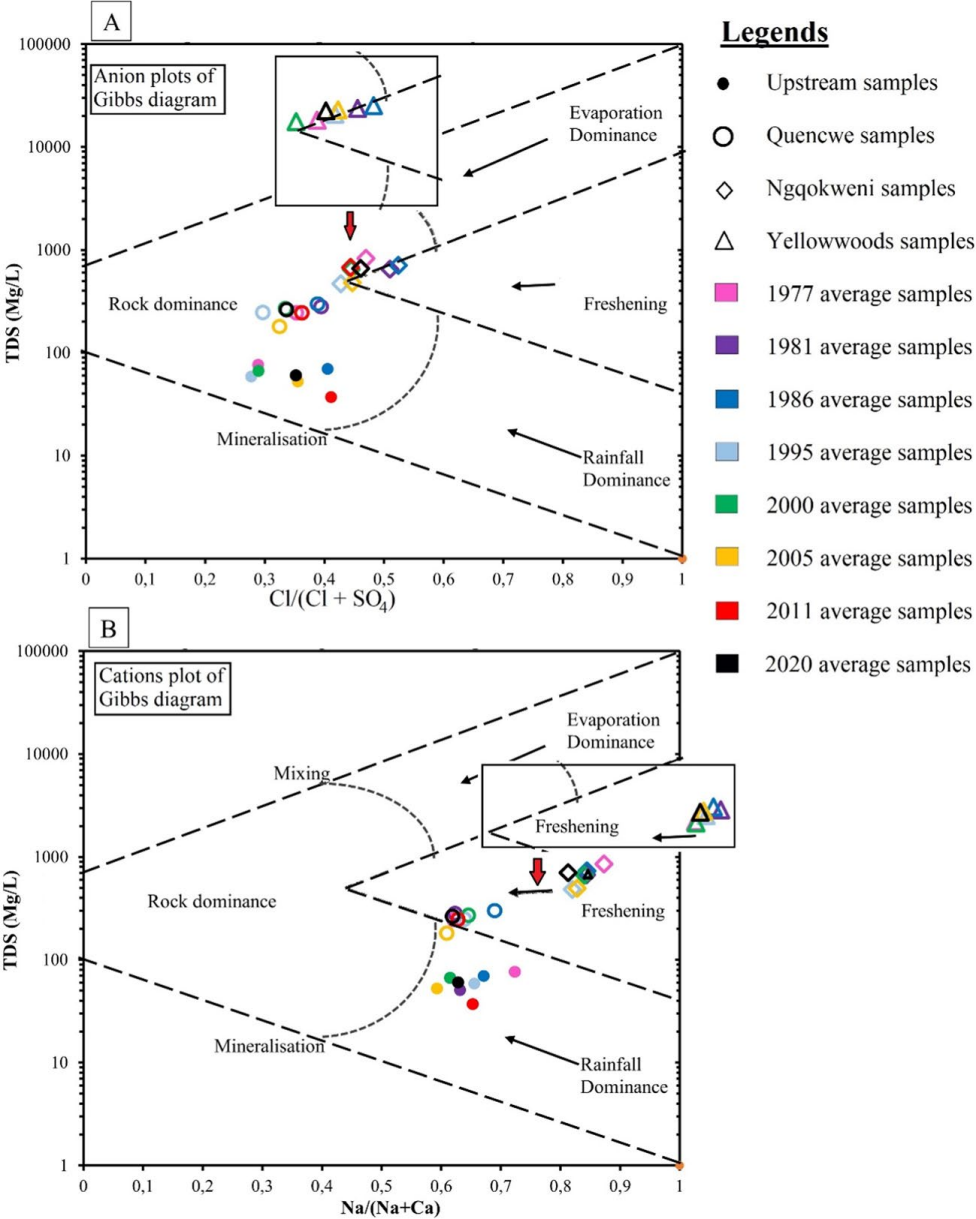
Gibbs plots showing water quality evolution (anions (**A**) and cations (**B**)) of samples obtained from Upstream, Quencwe, Ngqokweni, and Yellowwoods hydrometric stations

Gibbs plots are employed to corroborate the temporal variation in hydro-chemical processes in response to the EHE, as presented in Fig. 7 A and B. All the anions plot within the phase of rock geochemical interaction with surface water chemistry, thus suggesting the long-time water residence and the vulnerability of Buffalo River health to drought, although contrarily, the cation plots suggest otherwise, with the excess of calcium over total sodium in the river. This could be linked to the influence of the calcrete soil layer of the Balfour Formation on the river. Gibbs diagram varies for each station in the order of Upstream, Quencwe, Ngqokweni, and Yellowwoods basins, corresponding to the gradation in insufficient rainfall and increasing salinity of the basin landscape type.

The plots patterns exhibit trendy amalgamation, with the Upstream (from the lower right), Quencwe (from the right), Ngqokweni (towards the upper right), and Yellowwoods (towards the upper right) plots depicting rock dominance from the freshening/rainfall dominance, thus validating the sensitivity of BCH rivers to EHE as described by the SHDI, WC, and SMK.. Importantly, by identifying 1977, 1995, 2000, and 2005 with rock dominance, the Gibbs plots corroborate the sequential Mann–Kendall change points and the hydro-chemical variability induced by landscape variation.

## Discussion

### Water quality impact assessment of extreme hydrological events

This study focuses on the impact of extreme hydrological events (EHE) on water quality (WQ) within the specified temporal and spatial scales. Both the categorical and periodic regressors proved robust for the intended assessment with the support of sequential Mann–Kendall trend analysis. However, the categorical regression undertaken using the confusion matrices projects a weak gradient for the parameter values as the EHE phases increase, especially under the Kappa coefficient, thus hampering the result confidence. The 2-phase classifies into a regular flow and drought session, the 3-phase classifies into the La Nina, steady flow, and the El Nino phases, and the 5-phase divides the streamflow into lower flow units. Drawing from the confusion matrix projections, the drought part was captured as the most impactful section of the EHE, while further segregation of the hydrologic risk introduces WQ trend disruption. This suggests that the progressive impact of climate change will continuously aggravate WQ degradation as the flux in the hydrological cycle and water budget persist. In agreement, Alnahit et al. (2020) and Mishra et al. (2021) discovered that both the flood and drought phases of hydrologic extremes drive water quality distortion. Also, Chow et al. (2023) noted that both drought and post-drought concentration spiked high for the pesticide environmental quality standard of rivers at the far western side of the study region. The juxtaposition of the SHDI projection with WC assists with framing the impact of EHE on WQ in the time and frequency domain. Hence, confusion matrices proved to be an effective nonparametric statistical classifier, while wavelet coherence projected as an influential stochastic analyst. Overall, the assessment has provided critical information about the implication of climate change on water quality.

Evidence from ground-truthing validation and literature established that wastewater treatment plants (WWTP) are the primary point source pollution in the Yellowwoods basins. At the same time, leachates from agricultural activities contribute to a nutrient elevation in both Yellowwoods and Ngqokweni basins. Moreover, numerous studies have alluded to the significant impact of the effluent from the WWTP on the Buffalo River and the Yellowwoods tributary, not captured in the LULC assessment (Chigor et al. 2013; Edokpayi et al. 2017; Ohoro et al. 2021). Despite the LULC variation across the basins (from Upstream to Yellowwoods), the insignificant difference in confusion matrices suggests that the impact of EHE on water quality is the same, although with varying response. The average coherence of the wavelet co-movement further confirms the deductions from the overall accuracy and sensitivity analysis of confusion matrices. Hence, drawing from the significant impact of EHE on water quality and its suitability, the assessment aligns with the IPCC (Change 2014) and Borrelli et al. (2020) position on EHE being an event stressor of water quality.

### Sensitivity of surface water quality to EHE

The Buffalo river’s hydro-chemical sensitivity to EHE is, to a significant extent, capable of triggering an unsustainable alteration in the long term. The long-term water quality trend declivity shown by plots of SMK provides a qualitative suggestion of the general dependence of parameter mobility on flow constancy, corresponding to Sun et al. (2012)’s sensitivity analysis. Prolonged dryness recedes environmental flow (Owolabi et al. 2020a) and lowers concentration mobility and diffusion, hence the declining concentration at the hydrometric station (Alnahit et al. 2020; Mishra et al. 2021). In support of this, WC provides explicit periodic details on the variation in WQ sensitivity to EHE across the frequency and temporal domain, and confusion matrices provides a lumped sensitivity measure through the Kappa coefficient. The high sensitivity was depicted by the high power coherence, significant prograding in sequential Mann–Kendall, and Kappa coefficient (> 7%), with variations across the parameters and spatially.

Gibbs diagram exhibits the non-correlation of anions and cations. This could be due to the dissimilar hydrochemistry of groundwater influx during streamflow recession. Owolabi et al. (2020a) reported the inflow of groundwater during prolonged dryness and low flow from unconfined (calcrete and sandstone) and confined (dolerite) aquifers, mainly rich in calcite and calcium-plagioclase minerals. Huat et al. (2012) and Warren (2016) reported that calcretes are calcium-rich duricrusts, resulting from the precipitation of calcite from groundwater due to climatic fluctuations and prolonged dryness, peculiar to the arid and semi-arid environment. Watershed with soft sedimentary rocks like calcrete often exhibits dissolved ion mobilization (Alnahit et al. 2020). The high calcium and chloride nutrients that account for a different lateral position on the Gibbs diagram are linked with the area’s geology. The fluctuation in concentration is considerably significant for environmental instability and biodiversity and their ecological services.

Based on the highly sensitive variables, this study identified WQI, LI, and nitrate as the most suitable indicators for natural landscapes, semi and artificial landscapes, and the assessment of hydrologic flux, respectively. WQI’s suitability as a chemodynamical indicator in a natural landscape is due to its chemometric advantage of integrating numerous physicochemical parameters. Moreover, the inconsistency in change points strongly suggests the need for parameterization of the natural landscape water quality trend, to provide an accurate and all-inclusive chemometric prediction of the fate of river health mainly within the forested landscape. LI’s sensitivity and suitability for chemometric evaluation of hydrologic flux are due to its elevated loads during prolonged dryness and its indifference to spatial variability. The intermittency of nitrate in Quencwe, Ngqokweni, and Yellowwoods stations justified its sensitivity to hydrological flux. This can be linked to its conservation in runoff, which is an essential requirement for chemodynamics. The decreasing trends of nitrate at Quencwe, Ngqokweni, and Yellowwoods stations during the prolonged dryness and low flow could be due to excessive denitrification (Mishra et al. 2021). The increasing trend of nitrate in the Upstream could be due to abundant oxygen diffusion and the favorable conversion of ammonium to more nitrate in the natural landscape, as there are no anthropogenic sources of nitrate. This is in accordance with Han et al. (2020)’s report that high water levels and limited oxygen are essential conditions for the microbial nitrate reduction process.

### Spatial significance of EHE impact on water quality

The study engaged LULC dynamics as the spatial analyst for assessing the variation across spatial units. The central control on WQ response is expected to be influenced by area coverage of anthropogenic activities, urbanization, and exposure area to evaporation. While the research is limited to the extent of contaminant source causation, the deduction made here are mainly subject to the limited environmental concerns collated during ground-truthing validation and the information derived from LULC. The study reveals that the semi and artificial landscapes are relatively more stable to short-term EHE-WQ stress than the natural landscape, as captured by the limited change points and its consistency. The variation in water quality trends in the semi and the artificial landscape area is possibly due to variation in the proportion of point source and non-point source, in conformity to Wright et al. (2014). Peña-Guerrero et al. (2020) noted that there are occasional mixed responses from WQ, which could be linked to the obvious variation in urbanization and pollution point sources. The LULC assessment presents no significant evolution; hence, the eventual impact of EHE on WQ degradation is portrayed, although it is reasonable to assume an intensive degradation where urbanization and farming are enormously significant (Delpla et al. 2009).

### Implications and recommendations for the existing regional water resource pollution policy

Based on the discovery here, the impact of drought on water quality must be incorporated into the existing NEMA Act (2008) as one of the climate change issues requiring frantic attention as outlined in the Integrated Pollution and Waste Management Policy (IP&WM). The highlights of South Africa’s IP&WM policy in agreement with the United Nations Framework Convention on Climate Change were mainly on the regulation of pollution due to oil and nuclear energy, and the high sea, Antarctic Treaty, nature, and agricultural resource conservation with no mention of drought impact. Moreover, the water reform on regulation of abstraction should be sequestered based on watershed low-flow capacity at the catchment management level.

Due to the unfavorable impact of drought on water quality, measures on how to benchmark river low-flow threshold must be improved (Owolabi et al. 2020a). Also, a strict adherence to rational abstraction of water resources based on the low-flow threshold of dammed ephemeral rivers must be maintained. Water governance at all government levels is encouraged to consider the auditing of functional water infrastructure and replacement of leaky infrastructures at the consumers end, considering the additional economic implication of drought on the annual non-revenue water loss estimated at US$385 million in 2012 (Mckenzie et al. 2012). Effective enforcement against non-strategic effluent management and river channel dumping/dunghilling must be strengthened due to eutrophication and sedimentation risk which aggravate drought impacts (Herbig 2019). Strategic interventions must be developed to discourage livestock roaming/grazing around the upland badlands, eroded terrains, and riparian areas due to their impact on sedimentation and the acceleration of drought impacts in rural areas and suburbs (Wilkinson 2018). Aquifer recharge through inter-basin transfer to vulnerable watershed could also be encouraged for ephemeral rivers proximal to flash flood-prone watersheds to improve drought resilience. Water provision for dwellers within vulnerable watersheds should implement water scheme diversification, which includes the development of groundwater resources and long water transfer schemes. The LULC changes in BCH raise praiseworthy notes on the productive implementation of United Nations sustainable goals on afforestation policy to improve resilience against climate change. However, the overall diminution of rivers at the expense of other LULC growth across BCH demands improved environmental management decision-making considering its net impact on WQ sensitivity, especially at the upland, given the increasing trends of nitrate and WQI.

## Conclusion

The investigation of Buffalo Catchment Headwaters’ (BCH) water quality response to an extreme hydrological event (EHE) has provided some salient climate–related environmental impacts and substantial additions to the body of knowledge. In capturing the spatial variability of EHE across different landscapes, BCH was delineated to protect the natural, semi-natural, and artificial landscapes, thus demonstrating its suitability for the EHE-WQ correlation assessment. To this end, a one-at-a-time regional sensitivity approach based on categorical and periodic evaluation, using confusion matrices and wavelet coherence correlation, proved moderately reliable for the investigation. The framework provided an overview of the impact of climate change and EHE on water quality. Furthermore, the following essential deductions were made from the study:

- Climate change and the drought aspect of the extreme hydrological event have a substantial impact on water quality evolution.
- Extreme hydrological events are a principal long-term environmental flow nutrient modulator.
- The flux of water quality trends in forested landscape is mainly due to variable’s dependency on mobility and hydrologic process.
- WQI, LI, and nitrates were identified as suitable indicators for natural landscapes, semi and artificial landscape, and watershed hydro-chemometric flux.
- The Kappa coefficient of the confusion matrices proved valuable in bolstering the sensitivity and susceptibility of landscapes with high artificial land transformation, primarily due to the risk of numerous non-point sources.
- The Kappa coefficient (> 0.07) and sensitivity analysis (> 0.70) thresholds could portend a valuable metric for assessing drought severity in areas already at risk of highly potent contaminants, as observed in the Ngqokweni and Yellowwoods basins.
- Further hydrological drought impact on Buffalo catchment headwater could be hazardous given their deteriorating effects on the suitability of water for domestic and agricultural purposes and biodiversity.

In general, Kappa coefficients project weak gradients, which suggest that further statistical improvement is required to smooth the effect of stochasticity on the two parameters (EHE and WQ). Given this, future studies could attempt scenarios of regressive smoothing, to improve the EHE and WQ relationship. While the relationship between the streamflow variable and standard hydrological drought index is excellent to an absolute one, the drought phase may require an arithmetic adjustment of the value, thus affecting the authenticity of the trend for co-movement computation. Future studies may assess the error matrix associated with the adjustment of SHDI negative values while computing in the frequency-time domain. An integrated model can be developed to simulate the future trend of drought based on defined LULC scenarios and their impacts on the current trends of water quality. With the increasing rate of urbanization in several cities of South Africa and the current challenge of a green economy, standardizing the low-flow threshold for drought risk may become essential, to benchmark the limit of river resilience to the escalating effect of quality degradation.

## Data Availability

The datasets used and/or analyzed in the current study are provided with the submitted manuscripts and as suitable references.
